# Dual regulatory role of cyclic di-GMP via the DgcY/HhmR complex in salt response of *Halomonas hydrothermalis* Y2

**DOI:** 10.1128/mbio.01498-25

**Published:** 2025-08-06

**Authors:** Ye Zhang, Wenkai Wang, Yuanxiang Liu, Chongzhou Li, Jinyan Liu, Luhua Feng, Yan Gao, Yishu Peng, Wei Wang, Chunfang Li, Ping Xu, Chunyu Yang

**Affiliations:** 1 State Key Laboratory of Microbial Technology, Institute of Microbial Technology, Qingdao, People's Republic of China; 2 State Key Laboratory of Microbial Metabolism, Shanghai Jiao Tong University 553742 , Shanghai, People's Republic of China; McMaster University, Hamilton, Ontario, Canada

**Keywords:** c-di-GMP, diguanylate cyclase, feedback inhibition, LysR-type transcriptional regulator, protein interactions

## Abstract

Bis-(3′−5′)-cyclic dimeric guanosine monophosphate (c-di-GMP) is a key second messenger synthesized by diguanylate cyclases (DGCs) and regulates diverse bacterial behaviors. While many DGC modules are integrated within complex regulatory cassettes to ensure precise control of cellular processes, simpler DGC-containing cassettes are also widespread across bacteria, despite the regulative modes of their DGC activity remaining to be explored. In this study, we characterized a unique regulative network within a simple DGC cassette in *Halomonas hydrothermalis* Y2, DgcY, which comprises a six-transmembrane domain and a GGDEF domain lacking the canonical autoinhibitory site. In-frame deletion of *dgcY* significantly impaired the biofilm formation and strain growth under high-salt stress, particularly at lower temperatures. Notably, we uncovered a novel feedback inhibition mechanism in this strain, whereby c-di-GMP suppresses DgcY activity by facilitating the formation of a complex between DgcY and a LysR-type transcriptional regulator, HhmR. This interaction, mediated by the regulatory domain of HhmR, obviously reduced DgcY activity, especially in the presence of c-di-GMP. Functionally, the deletion of *hhmR* remarkably affected the swimming motility and biofilm formation of *H. hydrothermalis* Y2. Collectively, these findings revealed a collaborative feedback regulatory mode, in which c-di-GMP cooperates with a transcriptional regulator to modulate the DGC activity, thereby regulating signal transduction and bacterial adaptation to osmotic stress.

**IMPORTANCE:**

Generally, c-di-GMP exerts negative feedback on DGC activity by binding to the autoinhibitory site of DGCs or functioning as a downstream effector for transcriptional regulators. In this study, we revealed a previously unrecognized mode of feedback inhibition, in which c-di-GMP indirectly suppresses DgcY activity by promoting complex formation between DgcY and a LysR-type transcriptional regulator. Moreover, this DGC-associated network contributed to bacterial adaptation under osmotic stress at low temperatures, providing new insight into how c-di-GMP signaling pathways are integrated in the cellular osmotic regulation.

## INTRODUCTION

Bis-(3′−5′)-cyclic dimeric guanosine monophosphate (c-di-GMP) is a ubiquitous bacterial second messenger that regulates a wide range of cellular processes, including biofilm formation, motility, polysaccharide synthesis, virulence, and bacterial development and morphogenesis (1–4). Intracellular levels of c-di-GMP vary dynamically in response to internal and external environments, regulated by the diguanylate cyclases (DGCs) and c-di-GMP-specific phosphodiesterases (PDEs) (4, 5). DGCs catalyze the formation of c-di-GMP from two GTP molecules via dimer formation, while PDEs degrade it into either 5′-phosphoguanylyl-(3′−5′)-guanosine (pGpG) or GMP (5–8). Furthermore, c-di-GMP exerts its regulative function by binding to diverse downstream effectors, including riboswitches, DNA-binding proteins, catalytically inactive DGCs/PDEs, and PilZ domain-containing proteins (9–12). These multilayered regulatory networks enable bacteria to rapidly sense environmental changes, modulate intracellular c-di-GMP concentrations, and ultimately produce a coordinated phenotypic output (4).

Considerable research has revealed the “signal switch” function of N-terminal sensory domains that connect to the functional domains of DGCs or PDEs. These N-terminal sensory domains receive the environmental or host signals, including O_2_, NO, light, and chemicals, and thereby regulate the DGCs or PDEs activities (4, 13). GGDEF domains are frequently fused with diverse structural modules, such as Per-Arnt-SIM (PAS) domains (14), phosphorylation-related receptor domains (15), and the GAF domains (16). In addition to these sensory modules, transmembrane (TM) domains are also commonly found to be linked to GGDEF domains. In *Pseudomonas aeruginosa*, there are a total of 16 GGDEF domains connected to TM domains (17, 18). For instance, SadC is a functional DGC harboring a five-TM domain, and truncation of this TM domain impairs cell aggregation, biofilm formation, and bacterial motility (18); NicD senses the extracellular dispersion cues through its seven-TM domain (19). Beyond these potential membrane-associated DGCs in the c-di-GMP signal network, membrane-associated adenylyl cyclases (ACs), which catalyze the synthesis of cyclic adenosine monophosphate (cAMP), also account for a large proportion of ACs, underscoring the significance of these TM sensors in second messenger-mediated signal networks (20, 21). However, despite their widespread occurrence, the specific signals sensed by these TM domains and associated physiological functions remain largely unexplored.

In addition to the environmental sensing to initiate the c-di-GMP synthesis, the rate of c-di-GMP synthesis, that is, the regulation of DGC activity also needs to be tightly regulated to meet the intracellular conditions. Currently, the most common feedback inhibition mechanism involves the direct binding of c-di-GMP to the autoinhibitory site (I-site), which restricts the conformational changes of the GGDEF domain and inhibits its activity (22, 23). Beyond its canonical function in allosteric control of DGC activity, the I-site in GcbC also facilitates the interaction between GcbC and the characterized c-di-GMP receptor LapD (24). In *P. fluorescens*, GcbC produces c-di-GMP only in the presence of LapD, and formation of the LapD/GcbC complex enhances c-di-GMP synthesis. Similar DGC/protein complexes have also been identified in other c-di-GMP signaling networks, where they regulate DGC activity through physical interactions. For instance, the DGC activity of PdcA in *Burkholderia thailandensis* is repressed through interaction with the phosphate-accepting response regulator PdcC (25). Thus, physical interactions with regulatory partners may represent an alternative mode for modulating the DGC catalytic efficiency, despite the limited understanding of this mechanism.

In *Halomonas hydrothermalis* Y2, a strain isolated from the artificial alkaline environment of pulp mill wastewater (26), we identified a distinct DGC-encoding gene, *dgcY*, located within the *Doe* gene cluster associated with ectoine degradation. DgcY features a simple architecture comprising a six-transmembrane (TM) domain and an active GGDEF domain. Functional analysis revealed that DgcY contributes to osmotic stress tolerance at low temperatures in *H. hydrothermalis* Y2, with its TM domain playing a significant role in the DGC activity. Notably, DgcY lacks the canonical I-site typically involved in feedback inhibition in DGCs. Furthermore, we uncovered a novel regulatory mode in which a LysR-type transcriptional regulator, HhmR, represses the DgcY activity through direct physical interaction. Interestingly, the presence of c-di-GMP further promoted the complex formation of DgcY-HhmR and strengthened the repression.

## RESULTS

### Special location and domain architecture of *dgcY* in *H. hydrothermalis* Y2

**Fig 1:**
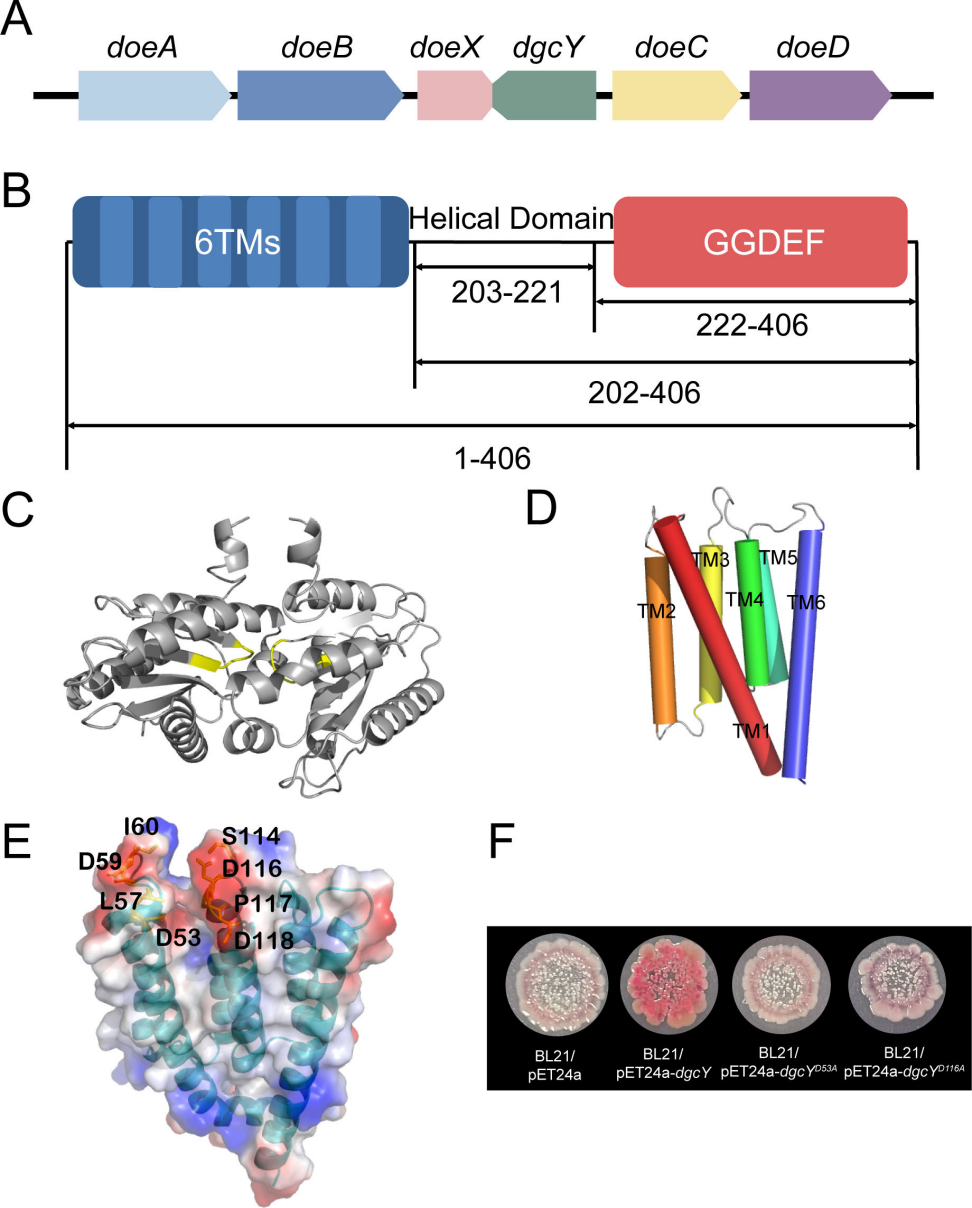
Genomic location, domain architecture, and structural model of DgcY. (**A**) The *dgcY* gene is located in the *Doe* gene cluster. (**B**) Predicted domain organization of DgcY by HMMTOP and SMART. (**C**) Predicted dimer structure of the intracellular fragment (DgcY_219–383_) by SWISS-MODEL with the template ImcA (PDB: 8WCN) (29). The GGDEF motif was colored in yellow. (**D**) Predicted monomer structure of the TM domain (DgcY_17–201_) by SWISS-MODEL with the template AC9 (PDB: 6R4O) (21). The TM1 to TM6 were labeled in black. (**E**) Surface representation of predicted DgcY_17–201_ monomer model using SWISS-MODEL, colored according to electrostatic potential. Low electrostatic potential energy to high electrostatic potential energy was colored from red to blue. The potential residues to form the EX site were labeled in black. (**F**) Congo red-binding ability of BL21/pET24a, BL21/pET24a-*dgcY*, BL21/pET24a-*dgcY^D53A^*, and BL21/pET24a-*dgcY^D116A^*.

The *Doe* gene cluster in *H. hydrothermalis* Y2 (accession no. SRP073747) contains six *Orfs* ranging from *Orf00344* to *Orf00349*. In this cluster, five putative genes are predicted to encode enzymes in an ectoine degradation pathway, including DoeA (ectoine hydrolase), DoeB (Na-acetyl-l-2,4-diaminobutyric acid deacetylase), DoeX (transcriptional regulator), DoeC (aspartate-semialdehyde dehydrogenase), and DoeD (l-2,4-diaminobutyric acid transaminase) (27). In our previous study on the construction of a high-yield ectoine-producing strain, deletion of *doeA* blocked the ectoine degradative pathway and remarkably enhanced its extracellular accumulation (28). Besides the five enzymes of the Doe pathway, we identified an additional *Orf* in this cluster, designated as *dgcY*, located between *doeX* and *doeC,* and overlapping with *doeX* in the reverse orientation (Fig. 1A). Notably, DgcY contains a GGDEF domain and was predicted as a membrane-associated protein. Topological structure analysis revealed that the intracellular C-terminal GGDEF domain (residues 222–406) is connected to an N-terminal 6-TM domain (residues 1–202) via a helical domain (HD; residues 203–221) (Fig. 1B).

Initially, we utilized AlphaFold3 to generate a dimeric model for the full-length DgcY fragment (Fig. S1A and B). The interface predicted TM-score (ipTM) value and predicted TM-score (pTM) values were 0.6 and 0.61, respectively. However, two GGEEF domains of the dimer were spatially distant and did not adopt the typical antiparallel conformation characteristic of the active state (Fig. S1C). Further modeling of the complete DgcY fragment in SWISS-MODEL was unsuccessful due to the lack of a homologue template. We then modeled the intracellular GGDEF domain and obtained a dimeric structure for DgcY_219–383_ (Fig. 1C), using the structure of ImcA of *P. aeruginosa* as the template (PDB: 8WCN; 80.0% coverage and 41.1% identity) (29). The resulting model adopted an active conformation, with a Global Model Quality Estimate (GMQE) score of 0.33 and a QMEANDisCo global score of 0.62 ± 0.05. By contrast, the predicted dimer structure of the intracellular fragment of DgcY (DgcY_219–383_) by AlphaFold3 again failed to adopt an active conformation (Fig. S1D to F). Therefore, the SWISS-MODEL-predicted structure was selected for subsequent docking and catalytic analyses.

Furthermore, the TM region (DgcY_1–201_) was predicted by AlphaFold3, yielding a structural model with a predicted Template modeling (pTM) score of 0.82 (Fig. S1G and H). Homology modeling via SWISS-MODEL unexpectedly selected adenylate cyclase 9 (AC9; PDB ID: 6R4O) as the template, which also contains six TM helices and shares 9.19% sequence identity with DgcY_1–201_ (Fig. 1D) (21). The resulting model exhibited a GMQE score of 0.46 and a QMEANDisCo global score of 0.49 ± 0.06, with 91.0% sequence coverage. In addition, the amino sequences of TM1 and TM2 in DgcY also shared 10.6% identity with those of another well-characterized adenylate cyclase, Cya (PDB: 7YZK) (20). The predicted structure was similar to that generated by AlphaFold3, with a Root Mean Square Deviation (RMSD) value of 3.54 Å. Based on the location of a negatively charged pocket (site Ex1) at the extracellular interface of Cya and its proposed function in recognizing positively charged ions or small molecules (20), we scanned the TM domain and identified several acidic residues, D53, D59, I60, D116, P117, and D118. These residues are highly conserved and positioned analogously to those in site Ex1 of Cya (Fig. 1E), specifically located at the two adjacent TM helices of DgcY on the outer side of the inner membrane of *H. hydrothermalis* Y2. Site-directed mutagenesis of D53 and D116 to Ala both abolished DgcY activity, with the appearance of white BL21/pET24a-*dgcY^D53A^* and BL21/pET24a-*dgcY^D116A^* colonies on Congo red plates (Fig. 1F). Together, these results suggested that the TM domain, especially its conserved acidic residues, is critical for DgcY activity in the signaling translocation pathway.

### Diguanylate cyclase activity of DgcY

**Fig 2:**
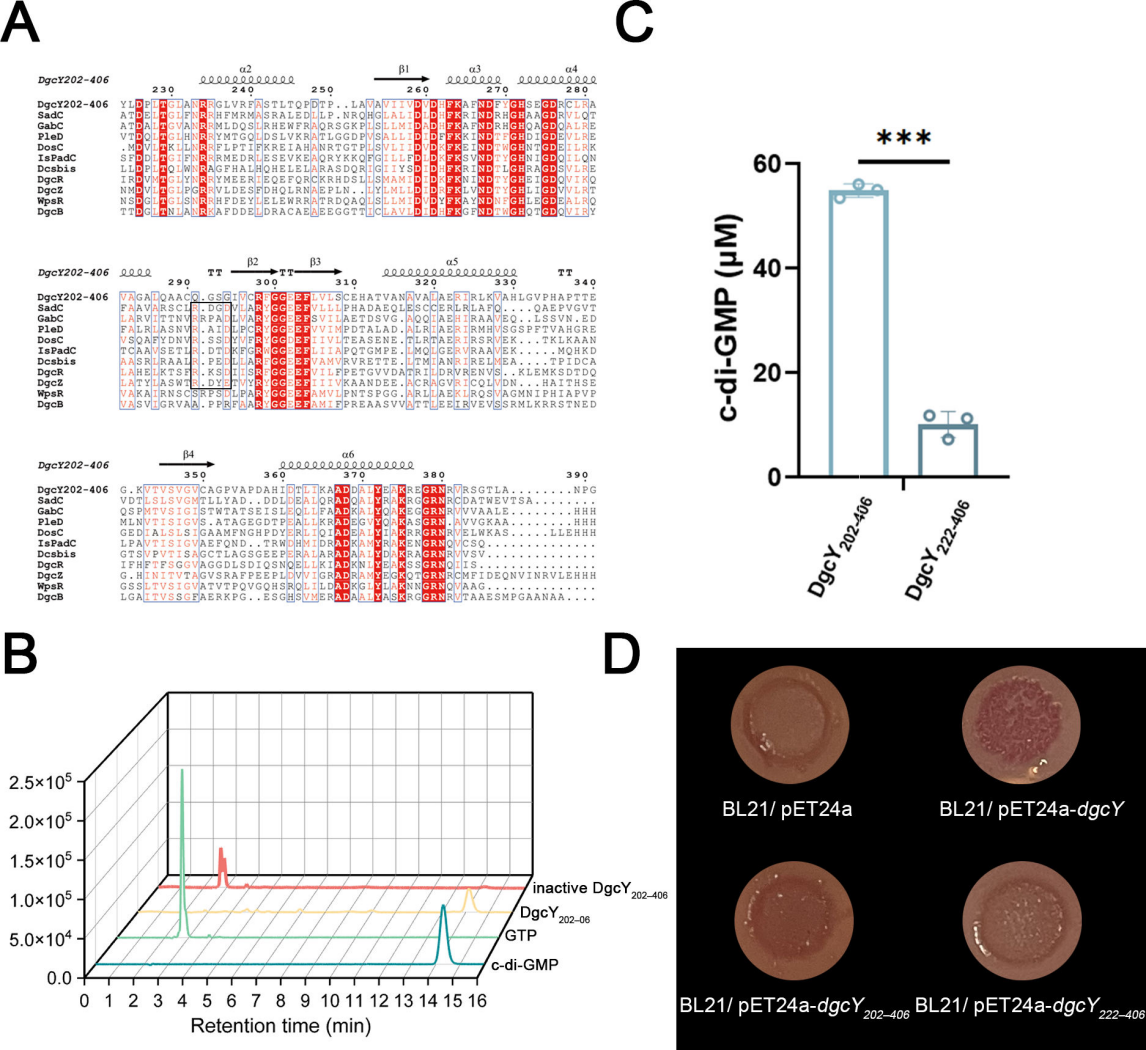
Sequence alignment, catalytic activity, and Congo red assay of DgcY_202–406_**.** (**A**) Sequence alignment of GGD(E)EF domain. Red background indicates highly conserved amino acids in the sequence alignment. The black box showed the conserved I-site (RXXD motif) in some DGCs. (**B**) *In vitro* catalytic activity of DgcY_202–406_ and inactive DgcY_202–406_. The c-di-GMP concentration was detected by high-performance liquid chromatography (HPLC). 2 mM GTP and 100 µM c-di-GMP were used as standards. (**C**) *In vitro* catalytic activity of purified DgcY_202–406_ and DgcY_222–406_ detected by HPLC. (**D**) Congo red-binding ability of BL21/pET24a, BL21/pET24a-*dgcY*, BL21/pET24a-*dgcY_202–406_*, and BL21/pET24a-*dgcY_222–406_*. Error bars show means and SD from triplicate experiments. Statistical significance was determined using T-tests. *P* values are denoted as * for *P* < 0.05, ** for *P* < 0.01, and ***for *P* < 0.001.

Multiple sequence alignments of DgcY with other homologs revealed a highly conserved GGD(E)EF motif, while the RXXD motif (I-site) for product feedback inhibition was absent (Fig. 2A). To assess its catalytic activity in c-di-GMP synthesis, the intracellular fragment, DgcY_202–406_, was heterologously expressed in *E. coli* BL21(DE3) (BL21/pET24a-*dgcY_202–406_*) and purified for DGC activity assay. As expected, DgcY_202–406_ exhibited high activity, while the heat-inactivated DgcY_202–406_ completely lost its ability to synthesize c-di-GMP (Fig. 2B). By contrast, the further truncated variant DgcY_222–406_, which, in the absence of the connecting HD region, exhibited markedly reduced catalytic activity (Fig. 2C). These results were consistent with previous observations on SadC, where the HD-containing fragment SadC_300–487_ promotes c-di-GMP production, whereas SadC_323–487_ is catalytically inactive. Structural analysis of SadC revealed that the HD fragment (300–322) may form an α-helix bundle within the dimer, and conformationally mediate the formation of an active oligomer for GTP catalysis (18).

Since c-di-GMP can promote polysaccharide production in *E. coli*, such as cellulose (30), we employed the Congo red-binding assay to evaluate the DGC activity of these constructs. As expected, BL21/pET24a-*dgcY_202–406_* formed red bacterial lawn on Congo red plates, whereas BL21/pET24a and BL21/pET24a-*dgcY_222–406_* produced orange-white lawns (Fig. 2D). In addition, aggregation assays showed that strain BL21/pET24a-*dgcY_202–406_* absorbs approximately twice as much Congo red as the control strain BL21/pET24a, indicating that the expression of DgcY_202–406_ prompted polysaccharide synthesis and accelerated aggregation (Fig. S2). These data confirmed that DgcY is functional in c-di-GMP synthesis and its associated phenotypes.

### DgcY regulates swimming motility, aggregation, polysaccharide synthesis, and biofilm formation in *H. hydrothermalis* Y2

**Fig 3:**
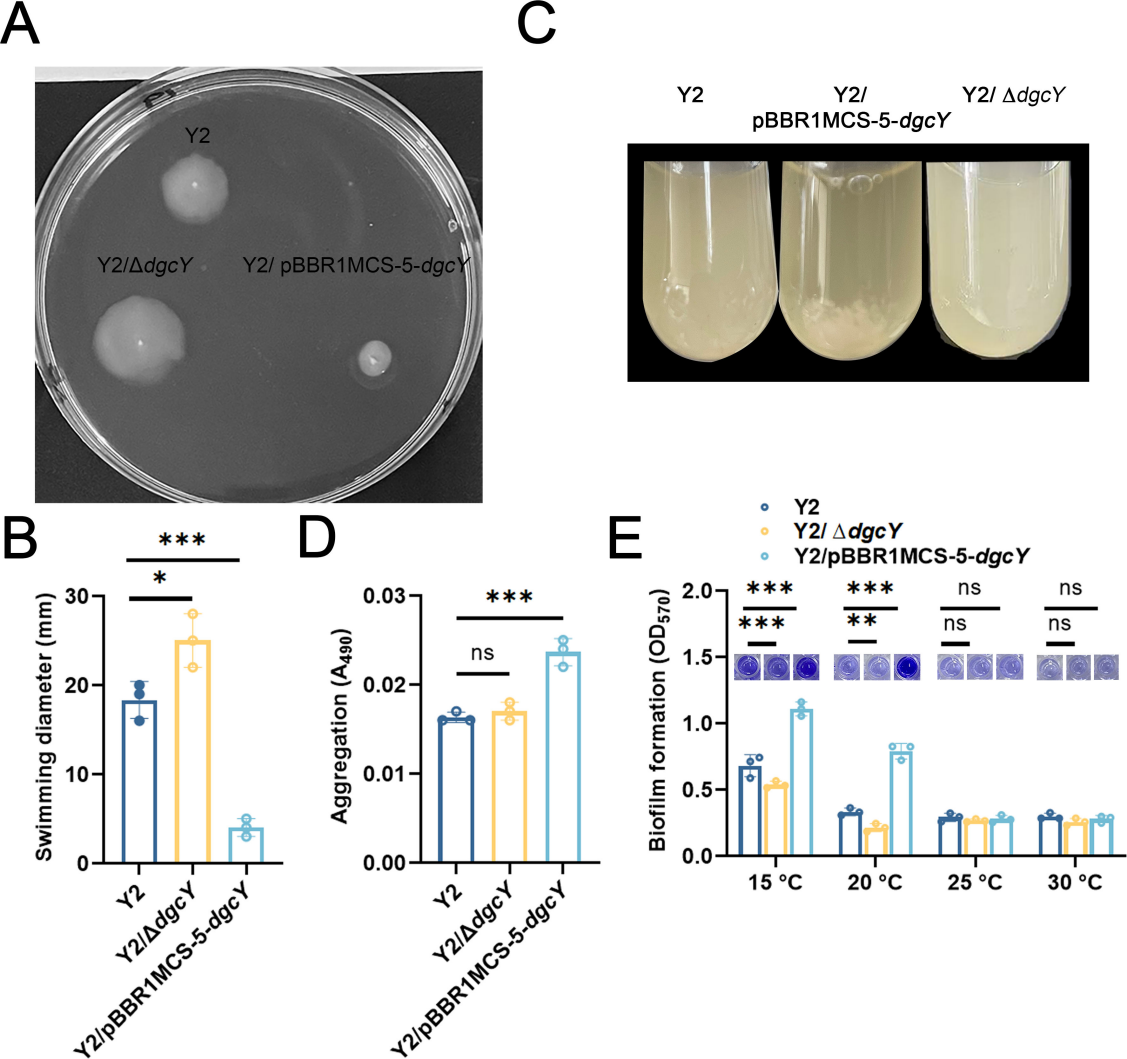
DgcY regulates swimming motility, aggregation, polysaccharide synthesis, and biofilm formation in *H. hydrothermalis* Y2. (**A and B**) Swimming motility of strain Y2, Y2/pBBR1MCS-5-*dgcY*, and Y2/Δ*dgcY* in 0.3% soft agar LB plates. (**C**) Cell aggregations of strain Y2, Y2/pBBR1MCS-5-*dgcY*, and Y2/Δ*dgcY* in LB culture. (**D**) Cell aggregations with Congo red binding of strain Y2, Y2/pBBR1MCS-5-*dgcY*, and Y2/Δ*dgcY* in M9 medium supplemented with Congo red. (**E**) Biofilm formation of strain Y2, Y2/pBBR1MCS-5-*dgcY*, and Y2/Δ*dgcY* in LB culture at different temperatures. Error bars show means and SD from triplicate experiments. Statistical significance was determined using a two-way ANOVA test. *P* values are denoted as * for *P* < 0.05, ** for *P* < 0.01, ***for *P* < 0.001,and ns for no significance.

Based on the observations that the heterologously expressed DgcY_202–406_ was catalytically active in *E. coli*, we further investigated the physical roles of DgcY in *H. hydrothermalis* Y2 by generating an in-frame *dgcY* deletion strain (Y2/Δ*dgcY*) and a *dgcY*-overexpressing strain (Y2/pBBR1MCS-5-*dgcY*). No difference was observed in the growth rates of strains Y2, Y2/Δ*dgcY*, and Y2/pBBR1MCS-5-*dgcY* when they were cultured in Luria–Bertani (LB) medium at 30°C (Fig. S3). We then investigated the swimming motility of these constructs in 0.3% soft agar LB plates and found that the motility of Y2/Δ*dgcY* was obviously higher than that of strain Y2, with the diameter of the swimming circle approximately 1.4 times greater than that of strain Y2 (Fig. 3A and B). More notably, the swimming circle diameter of Y2/pBBR1MCS5-*dgcY* was significantly reduced to approximately 20% compared to that of strain Y2, suggesting that DgcY plays a role in regulating swimming motility in strain Y2. Consistently, the aggregation assay showed that DgcY overexpression led to an apparent cell aggregation, whereas no visible aggregation was observed in the other strains (Fig. 3C). When comparing the Congo red-binding abilities of these constructs, we found that DgcY overexpression resulted in significantly higher aggregation and Congo red-binding compared to that of wild-type Y2 (Fig. 3D), confirming that DgcY promotes extracellular polysaccharide synthesis in *H. hydrothermalis* Y2. By contrast, no obvious variation in extracellular polysaccharide level was observed in Y2/Δ*dgcY*. An explanation for such invariability could be that multiple proteins are involved in the regulation of polysaccharide production in strain Y2, such that the absence of DgcY alone does not lead to a substantial reduction in polysaccharide content.

When comparing the biofilm-formation ability of these strains, we observed no biofilm formation at 30°C (Fig. 3E). Since strain Y2 grows across a wide temperature range, from 4°C to 45°C (31), we cultured these strains at different temperatures and observed visible biofilms only at low temperatures (15°C and 20°C, Fig. 3E). Interestingly, overexpression or the absence of DgcY had no significant influence on biofilm formation compared to strain Y2 at 25°C and 30°C. However, as the temperature decreased to 20°C and 15°C, strain Y2/pBBR1MCS-5-*dgcY* displayed the highest biofilm-forming capacity, which was 1.7- and 2.3-fold higher than that of Y2, respectively. By contrast, nearly no biofilm was observed in Y2/Δ*dgcY* at 20°C and 15°C. Therefore, we suspected that DgcY may play a significant role in biofilm formation in *H. hydrothermalis* Y2 at low temperatures.

### DgcY plays an important role in resistance to osmotic stress caused by inorganic salts at low temperatures

**Fig 4:**
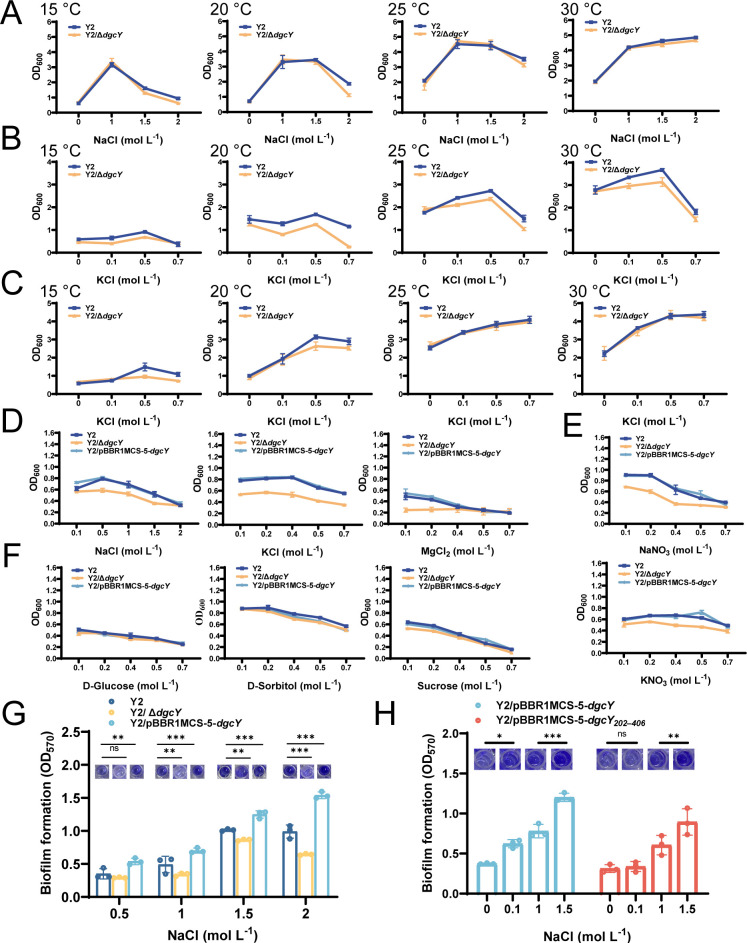
Growth of *H. hydrothermalis* Y2, Y2/pBBR1MCS-5-*dgcY*, and Y2/Δ*dgcY* under various stresses caused by inorganic and organic solutes. LB0 medium, absent of NaCl, was used as a basal medium for strain cultivation. (**A**) Strain growth in the shake tubes containing 5 mL LB0 medium with various concentrations of NaCl, cultured at different temperatures (from left to right: 15°C, 20°C, 25°C, and 30°C). (**B**) The same as (**A**) except using various concentrations of KCl. (**C**) The same as (**B**) except 1 g L^−1^ NaCl was added to LB0 media. (**D**) Strain growth in LB0 medium with different chlorinated salts, including NaCl, KCl, and MgCl_2_, at 15°C in the 96-well plates. (**E**) The same as (**D**) except using various concentrations of NaNO_3_ and KNO_3_. (**F**) The same as (**D**) except using D-Glucose, D-Sorbitol, and sucrose. (**G**) Biofilm formation of *H. hydrothermalis* Y2, Y2/Δ*dgcY*, and Y2/pBBR1MCS-5-*dgcY* in LB0 medium containing different concentrations of NaCl at 15°C. (**H**) Biofilm formation of Y2/pBBR1MCS-5-*dgcY*, and Y2/pBBR1MCS-5-*dgcY_202–406_* in LB0 medium containing different concentrations of NaCl at 15°C. Error bars show means and SD from triplicate experiments. Statistical significance was determined using a two-way ANOVA test. *P* values are denoted as * for *P* < 0.05, ** for *P* < 0.01, ***for *P* < 0.001, and ns for no significance.

As described, *dgcY* is located in the *Doe* gene cluster that is responsible for ectoine degradation (27). As a halotolerant and ectoine-producing strain, strain Y2 requires a certain concentration of Na^+^ for optimal growth (Fig. 4A), but it is sensitive to high NaCl concentrations (28). We then investigated the growth of Y2 and Y2/Δ*dgcY* at LB0 medium (LB medium without NaCl addition) with different NaCl concentrations in the shake tubes. As shown in Fig. 4A, both strains exhibited similar growth profiles at 25°C and 30°C. However, at 15°C and 20°C, the growth of Y2/Δ*dgcY* was obviously inhibited in the presence of 2.0 M NaCl, with the optical density values (OD_600_) reduced by approximately 40% compared to that of Y2. Meanwhile, there was no significant difference in growth between Y2 and Y2/Δ*dgcY* in LB0 medium, verifying that the impaired growth of Y2/Δ*dgcY* is likely due to the stress caused by high Na^+^ concentrations (Fig. 4A). Compared to its sensitivity to NaCl, Y2/Δ*dgcY* was more sensitive to KCl, with growth being significantly inhibited even by 0.1 M KCl at 15°C, 20°C, and 25°C (Fig. 4B). Our previous study showed that when exposed to high external K^+^ concentrations, strain Y2 extrudes excess intracellular K^+^ with the assistance of Na^+^ (32). We therefore supplemented 1 g L^−1^ NaCl to the LB0 medium and investigated the K^+^ sensitivity again. Consistent with our previous findings, improved growth was observed in all cultures (Fig. 4C). Notably, Y2/Δ*dgcY* became less sensitive to K^+^ stress, with OD_600_ values comparable to those of strain Y2 at 25°C and 30°C (Fig. 4C). However, similar to the low-temperature growth profiles under NaCl stress, the growth of Y2/Δ*dgcY* was still inhibited at 15°C and 20°C under conditions of 1 g L^−1^ NaCl and 0.5 M or 0.7 M KCl (Fig. 4C). These results suggested that DgcY plays an important role in coupling with high Na^+^ or K^+^ toxicity.

By selecting a lower temperature of 15°C as the cultivation condition and culturing in the 96-well plates, we further compared the growth profiles of strains Y2, Y2/Δ*dgcY*, and the *dgcY*-overexpressed strain Y2/pBBR1MCS-5-*dgcY* under various inorganic salts and organic compounds. Worth noticing, all strains showed enhanced salt sensitivity in the 96-well plates compared to those in the shake tubes (Fig. 4A to through C). We hypothesized that limited dissolved oxygen in the 96-well plates imposes additional stress on the cells and leads to such enhanced sensitivity. As presented in Fig. 4D, strain Y2 and Y2/pBBR1MCS-5-*dgcY* exhibited similar growth profiles in various NaCl-containing media, with Y2/pBBR1MCS-5-*dgcY* growing better in the presence of a low NaCl concentration, specifically 0.1 M. By contrast, the growth of Y2/Δ*dgcY* was seriously inhibited by 0.5–1.5 M NaCl. Consistent with the growth profile in NaCl-supplemented medium, strain Y2/Δ*dgcY* exhibited impaired growth under the tested concentrations of KCl and MgCl₂, with OD_600_ values approximately 0.2-fold lower than those of strain Y2. Moreover, strain Y2/Δ*dgcY* was also sensitive to NaNO_3_ and KNO_3_, with obviously reduced growth compared to that of the wild-type strain (Fig. 4E). In contrast to the observed growth difference in the presence of these inorganic ions, the growth of Y2/Δ*dgcY* showed nearly no changes in the presence of various concentrations of D-glucose, D-sorbitol, and sucrose (Fig. 4F). Therefore, we concluded that DgcY plays an indispensable role in resistance to osmotic stress caused by inorganic salts.

Given the increased salt sensitivity of the DgcY-deficient strain, we hypothesized that DgcY plays a critical role in resistance to inorganic salt stress. For further confirmation, we compared biofilm formation among three strains under different NaCl concentrations at 15°C. Notably, Y2/pBBR1MCS-5-*dgcY* exhibited the highest biofilm formation, which sharply increased under 2 M NaCl (Fig. 4G). By contrast, both the wild-type strain and Y2/Δ*dgcY* showed reduced biofilm formation at 2 M NaCl compared to 1.5 M NaCl (Fig. 4G). To evaluate the functions of the N-terminal six-TM domain of DgcY, we compared the biofilm formation capacity of Y2/pBBR1MCS-5-*dgcY* with a construct carrying only the intracellular portion DgcY (Y2/pBBR1MCS-5-*dgcY_202–406_*). As shown in Fig. 4H, truncation of the TM domain significantly impaired the biofilm formation under all tested salt concentrations. This phenotype was also consistent with impaired swimming motility (Fig. S4A and B), further confirming the indispensable function of the TM domain in DgcY activity. Notably, compared to the LB0 medium, the addition of 0.1 M NaCl remarkably increased the biofilm formation of strain Y2/pBBR1MCS-5-*dgcY*, while no variation was observed in Y2/pBBR1MCS-5-*dgcY_202-406_*. Subsequently, when the NaCl concentration increased from 1.0 to 1.5 M, more biofilm was produced in strain Y2/pBBR1MCS-5-*dgcY* (Fig. 4H). These results suggested that the TM domain is essential in NaCl-related biofilm formation and the function of DgcY.

### DgcY_202–406_ interacts with the transcriptional regulator HhmR

**Fig 5:**
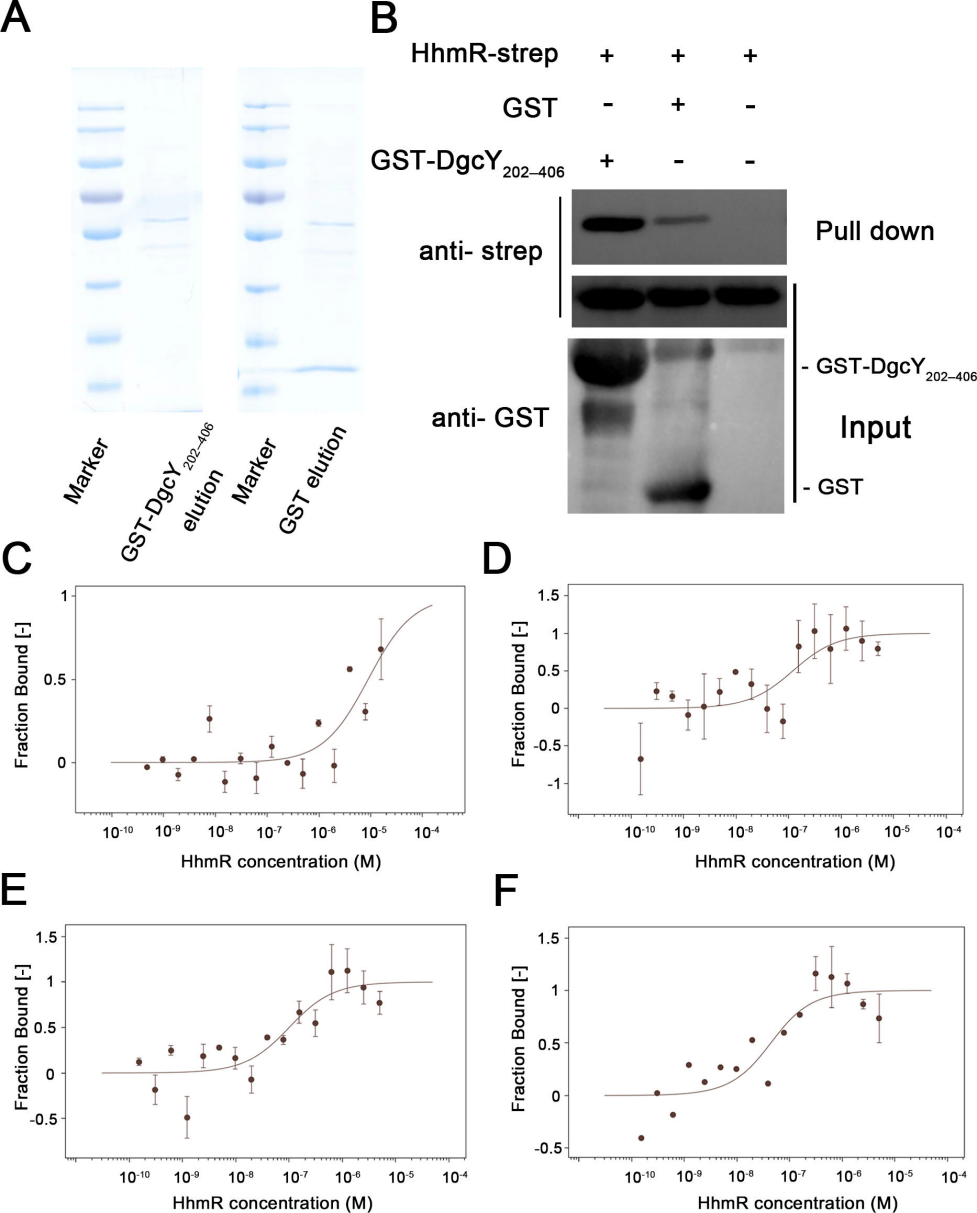
DgcY_202–406_ interacts with HhmR. (**A**) SDS-PAGE spectrum of the GST pull-down elution samples. (**B**) GST pull-down and western blot assays confirmed the interaction between DgcY_202–406_ and HhmR. Rosetta/pET24a-*hhmR-strep* cell lysis was incubated with GST or GST-DgcY_202–406_ fused resins, and the interaction complexes were eluted for western blot assays. (**C**) Microscale thermophoresis (MST) assays showed DgcY_202–406_ binds to HhmR, and the *K_D_* was 8.96 µM. (**D**) MST assays showed DgcY_202–406_ binds to HhmR in the buffer containing 0.5 µM c‐di‐GMP, and *K_D_* was 104.73 nM. (**E**) MST assays showed DgcY_202–406_ binds to HhmR in the buffer containing 2.5 µM c‐di‐GMP, and *K_D_* was 90.49 nM. (**F**) MST assays showed DgcY_202–406_ bound to HhmR in the buffer containing 15 µM c‐di‐GMP, and *K_D_* was 34.06 nM. Error bars show means and SD from triplicate experiments.

It has been proposed that proteins in the c-di-GMP signaling pathway generally form “microcompartments,” where DGCs and PDEs function as central elements by interacting with other proteins to regulate intracellular c-di-GMP levels (33–35). Given the absence of an I-site in the DgcY, we explored potential DgcY-interacting partners in strain Y2. A GST-fusion protein, GST-DgcY_202–406_, was expressed in *E. coli* BL21 (DE3) and purified for Glutathione-S-transferase pulldown (GST pull-down) assays (Fig. 5A). Among proteins identified by Q Exactive LC/MS analyses (Tables S1 and S2), a transcriptional regulator (HhmR) belonging to the LysR-type transcriptional regulator (LTTR) family was of particular interest, for its recorded functions in biofilm formation, cell motility, and extracellular polysaccharide production (36, 37).

To confirm the *in vitro* interaction between HhmR and DgcY_202–406_, *E. coli* strain Rosetta/pET24a-*hhmR-strep* was constructed for GST pull-down. As expected, a distinct band corresponding to HhmR-GST-DgcY_202–406_ binding was observed in the GST pull-down and western blot analyses (Fig. 5B), along with the presence of HhmR detected by Q Exactive LC/MS spectrum (Table S3). Subsequently, we employed microscale thermophoresis (MST) to measure the binding affinity between DgcY_202–406_ and HhmR. Titration of purified DgcY_202–406_ with serial dilutions of HhmR yielded a dissociation constant (*K*_D_) of 8.96 µM (Fig. 5C), indicating a relatively strong interaction. To determine whether DgcY_202–406_ specifically interacts with HhmR or also binds other LTTR-family proteins, we scanned the Y2 genome and identified 32 LTTR family regulators. The closest homolog of HhmR, sharing 31.2% sequence identity, was selected for binding affinity analysis. However, no interaction between the homologue and DgcY_202–406_ was detected in the GST pull-down and western blot assays (Fig. S5A), suggesting that the interaction between HhmR and DgcY_202–406_ represents a specific regulatory mechanism.

### HhmR plays a c-di-GMP-dependent regulatory role in the DgcY-related signaling pathway

**Fig 6:**
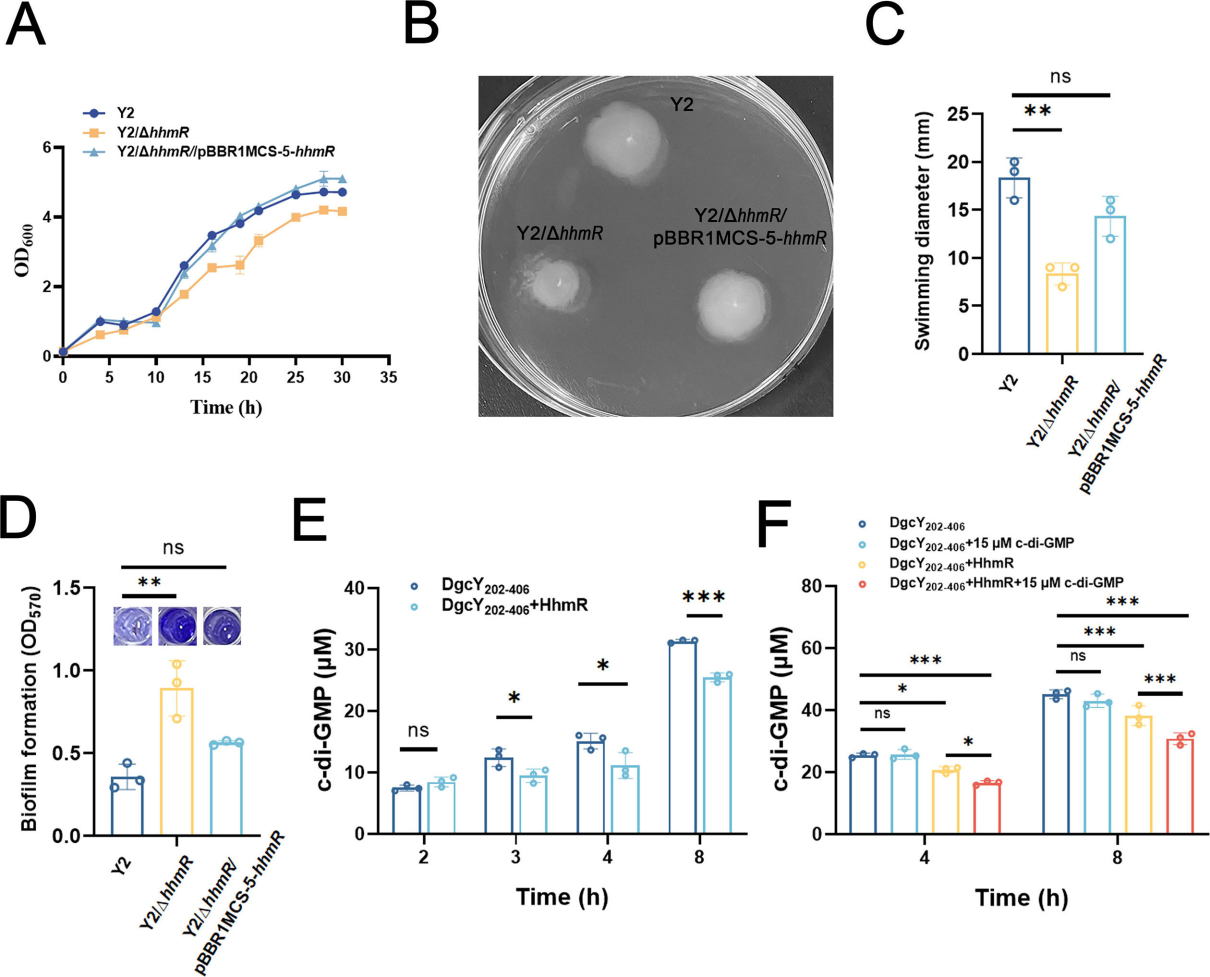
Influence of HhmR on the DgcY_202–406_ activity and cell behaviors of *H. hydrothermalis* Y2. (**A**) Growth curves of strain Y2, Y2/Δ*hhmR*, and Y2/Δ*hhmR*/pBBR1MCS-5-*hhmR* in LB medium and cultured at 30°C, 200 rpm. (**B and C**) Swimming motility of strain Y2, Y2/Δ*hhmR*, and Y2/Δ*hhmR*/pBBR1MCS-5-*hhmR* in 0.3% soft agar LB plates. (**D**) Biofilm formation of strain Y2, Y2/Δ*hhmR*, and Y2/Δ*hhmR*/pBBR1MCS-5-*hhmR* in LB culture at 15°C. (**E**) The c-di-GMP concentration in the reaction mixture of DgcY_202–406_ in supplementation with or without HhmR was detected by HPLC. Incubation was performed at 37°C for 2 h, 3 h, 4 h, and 8 h. (**F**) The c-di-GMP concentration in the reaction mixture of DgcY_202–406_ with or without HhmR and (or) c-di-GMP addition was detected by HPLC. Incubation was performed at 37°C for 4 h and 8 h. Error bars show means and SD from triplicate experiments. Statistical significance was determined using a two-way ANOVA test. *P* values are denoted as * for *P* < 0.05, ** for *P* < 0.01, ***for *P* < 0.001, and ns for not significant.

To investigate the physiological role of HhmR in the DgcY-associated signaling pathway, we deleted the *hhmR* gene from the genome of strain Y2 and assessed related phenotypes. Compared to strain Y2, the growth rate of Y2/Δ*hhmR* was significantly reduced during the exponential phase at 30°C, which could be restored to wild-type levels upon complementation with plasmid-borne *hhmR* (Y2/Δ*hhmR*/pBBR1MCS-5-*hhmR*) (Fig. 6A). Strain Y2/Δ*hhmR* also showed apparently impaired swimming motility, with a half-fold reduced swimming diameter detected (Fig. 6B and C). As expected, the swimming diameter of Y2/Δ*hhmR*/pBBR1MCS-5-*hhmR* was restored to a level identical to that of Y2. Consistently, compared to strain Y2, the biofilm formation of Y2/Δ*hhmR* was approximately twofold higher at 15°C and was reduced again to the ordinary level in the complementary strain (Fig. 6D). These results confirmed that HhmR is involved in regulating swimming motility and biofilm formation in strain Y2, acting as a negative regulator within the c-di-GMP signaling pathway.

Next, we determined whether HhmR regulates the catalytic activity of DgcY_202–406_ by adding purified HhmR to an *in vitro* reaction mixture or not. As shown in Fig. 6E, no difference in c-di-GMP concentration was observed between the two reaction systems during the initial 2 h reaction. However, after 3 h of reaction, the reaction mixture lacking HhmR produced 12.41 µM c-di-GMP, whereas the co-incubation mixture yielded only 9.47 µM (Fig. 6E), indicating that HhmR attenuates the DgcY catalytic activity. Also, this repressive effect lasted during the reaction, with 31.38 and 25.50 µM c-di-GMP detected in the respective reactions after 8 h incubation. Together, these data suggested that HhmR negatively affects the DgcY-mediated c-di-GMP synthesis in strain Y2, which may subsequently regulate the c-di-GMP level in the “local pool” and thereby influence the biophysical functions of this strain.

In the c-di-GMP signaling pathway, transcriptional regulators are often identified as c-di-GMP effectors that regulate downstream gene expression by binding to c-di-GMP. To investigate the cues associated with c-di-GMP in the DgcY_202–406_-HhmR cascade, we used MST assays to measure the binding affinities of these three components. Unexpectedly, no binding was detected between HhmR and c-di-GMP (Fig. S5B). In addition, the interaction between c-di-GMP and DgcY_202–406_ exhibited a *K_D_* value of 53.25 µM (Fig. S5C), suggesting that c-di-GMP may bind to the active site of DgcY or to other regions that mediate feedback inhibition, similar to the I-site. Interestingly, the binding affinity between DgcY_202–406_ and HhmR was positively proportional to the c-di-GMP concentration. In the buffer containing 0.5 or 2.5 µM c‐di‐GMP, the binding affinity between DgcY_202–406_ and HhmR was increased by approximately 100‐fold (Fig. 5D and E, *K_D_* of 104.73 or 90.49 nM, respectively), when compared to that of lacking c‐di‐GMP (*K_D_* of 8.96 µM, Fig. 5C). Meanwhile, in the presence of 15 µM c‐di‐GMP, the binding affinity of DgcY_202–406_ and HhmR was increased by approximately 263‐fold (Fig. 5F, *K_D_* of 34.06 nM). This indicated that c-di-GMP is involved in the DgcY_202–406_–HhmR interaction and functions as a binding promoter. In addition, due to the remarkably lower *K*_D_ values observed for the DgcY/HhmR complex, the binding energies from c-di-GMP and DgcY_202–406_ can be excluded in the three-component system. This supported the conclusion that c-di-GMP promotes the formation of DgcY/HhmR interaction. Compared to DgcY_202–406_, DgcY_222–406_, which absent of the HD region, exhibited a significantly decreased binding affinity with c-di-GMP (Fig. S5D, *K_D_* of 1.17 mM), indicating that the HD region plays a key role in the binding between c-di-GMP, DgcY, and HhmR.

To determine the synergistic effect of c-di-GMP and HhmR on the DGC activity of DgcY_202–406_, we added 15 µM c‐di‐GMP to the *in vitro* reaction mixture, which contains purified DgcY_202–406_ and HhmR. As expected, compared to the 38 µM c-di-GMP produced in reaction without additional c-di-GMP supplementation, the reaction mixture containing DgcY_202–406_, HhmR, and 15 µM c-di-GMP only produced 30 µM c-di-GMP after 8 h of reaction (Fig. 6F), indicating a synergistic inhibition between HhmR and c-di-GMP. By contrast, the addition of 15 µM c-di-GMP alone had no significant influence on DGC activity. Considering these *in vitro* reactions and MST data, we concluded that the regulatory effect of HhmR on the DgcY activity is mediated by its physical interaction with DgcY_202–406_, and c-di-GMP further intensifies this negative regulation.

### The regulatory domain (RD) of HhmR binds to DgcY_202–406_ to regulate its activity

**Fig 7:**
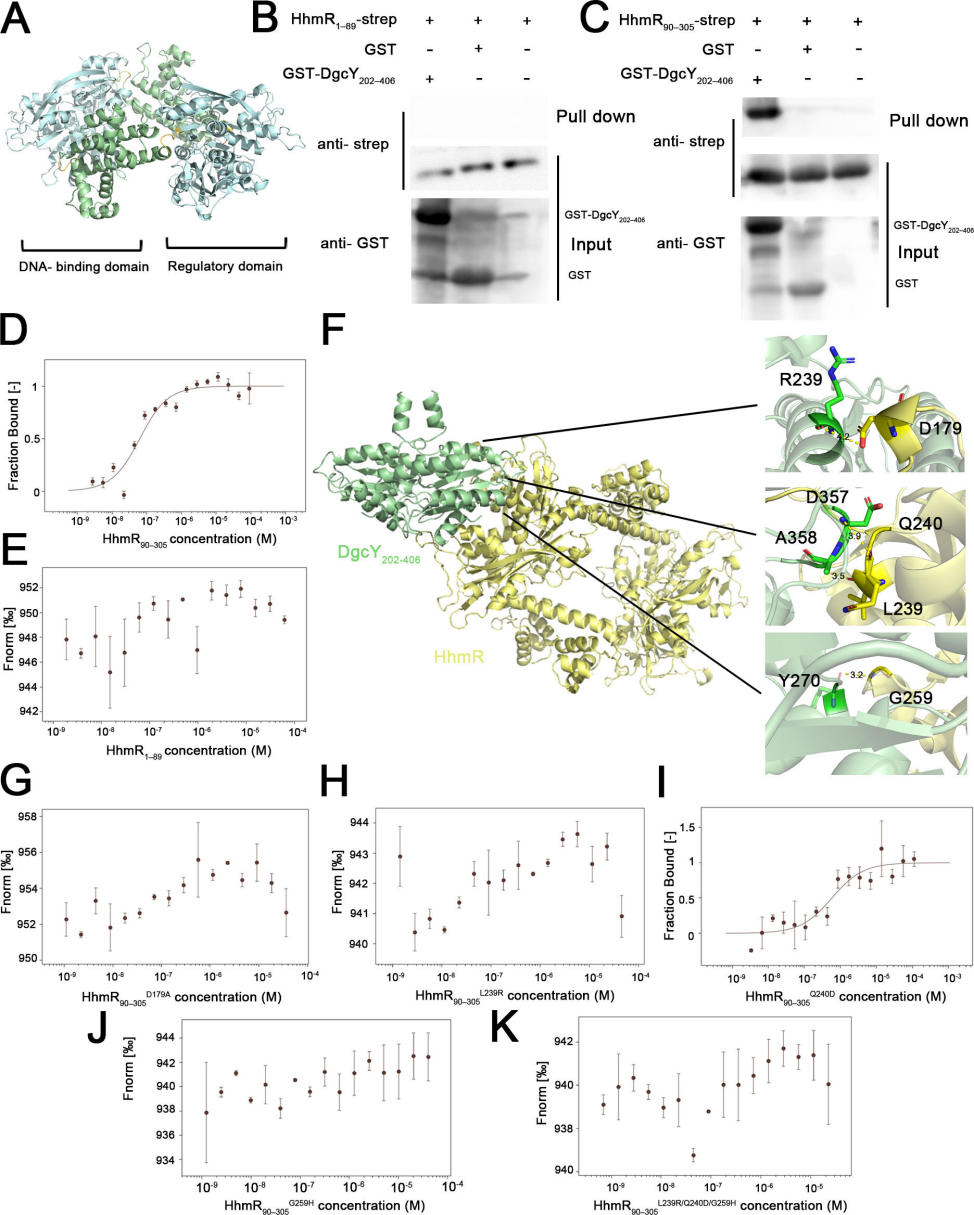
DgcY_202–406_ interacts with the RD domain of HhmR. (**A**) Predicted tetrameric structure of HhmR by SWISS-MODEL using the template DarR (PDB: 7DWN)(38). The DNA-binding domain was colored in green, the regulatory domain was colored in cyan, and the connected hinge was colored in yellow. (**B and C**) GST pull-down and western blot assays for the interaction of DgcY_202–406_ with HhmR_1–89_ or HhmR_90–305_. Rosetta/pET24a-*hhmR_1–89_-strep* or Rosetta/pET24a-*hhmR_90–305_-strep* cell lysis was incubated with GST or GST-DgcY_202–406_ fused resins, respectively, and the interaction complexes were eluted for western blot assays. (**D and E**) MST assays showed DgcY_202–406_ binds to HhmR_90–305_, and the *K_D_* value was 53.03 nM. There was no binding between DgcY_202–406_ and HhmR_1–89_. (**F**) The docking model of DgcY_202–406_ dimer with HhmR tetramer predicted by ZDOCK. The predicted binding regions of DgcY_202–406_ and HhmR were displayed in more detail on the right. (**G–K**) MST assays showed the binding affinity of DgcY_202–406_ with HhmR mutants. HhmR_90–305_^Q240D^ showed a significantly decreased binding affinity with DgcY_202–406_, and *K*_D_ was 570.71 nm. The MST assays showed no binding of DgcY_202–406_ with HhmR_90–305_^D179A^, HhmR_90–305_^L239R^, HhmR_90–305_^G259H^, and HhmR_90–305_^L239R /Q240D/G259H^.

To investigate the mechanism underlying the regulatory effect of HhmR on the DGC activity of DgcY, we predicted the tetrameric structure of HhmR using AlphaFold3 and SWISS-MODEL, respectively. The structure predicted by AlphaFold3 showed a compact conformation, with the ipTM value of 0.3 and pTM value of 0.4 (Fig. S6A and B). Differently, SWISS-MODEL generated a structure with an open conformation, based on the tetrameric structure of the LTTR family regulator DarR (PDB: 7DWN) in effector binding state (38). The GMQE value was 0.7, and the QMEANDisCo global score was 0.66 ± 0.05, with 95.0% coverage and 34.3% amino acid sequence identity. Several resolved structures of LTTR family proteins reveal a sliding dimer hypothesis, in which the effector binding induces an open conformation in LTTR proteins (39, 40). Regarding the direct repression of HhmR on the DGC activity, we hypothesized that HhmR may be recruited to DgcY upon inducer binding, thereby enabling its spatial interaction and subsequent negative regulation of DGC activity. Thus, the SWISS-MODEL structure with an open conformation would be plausible for identifying the potential binding region in HhmR. Consistent with other LTTR family regulators (38), the predicted HhmR structure consisted of a typical N-terminal DNA-binding domain (DBD) and a C-terminal regulatory domain, connected by a flexible hinge region (Fig. 7A). ZDOCK 2.3.2 analysis further predicted that the DgcY_202–406_ fragment primarily interacts with the RD region. To experimentally ascertain the interaction, truncation variants of HhmR, DBD (HhmR_1–89_), and RD (HhmR_90–305_) were, respectively, expressed in *E. coli* Rosetta (DE3) strains and purified for binding assays with DgcY_202–406_. Apparently, no detectable interaction was observed between GST-DgcY_202–406_ and HhmR_1–89_ through GST pull-down and western blot assays (Fig. 7B), whereas obvious interaction between GST-DgcY_202–406_ and HhmR_90–305_ was detected (Fig. 7C). Consistently, MST experiments showed that DgcY_202–406_ binds tightly to HhmR_90–305_ (Fig. 7D, *K_D_* of 53.03 nM), whereas no binding was observed with HhmR_1–89_ (Fig. 7E). Furthermore, in comparison to the relatively low binding affinity between DgcY_202–406_ and full-length HhmR (8.96 µM), we hypothesize that the N-terminal domain of HhmR may cause a conformational change that hinders its regulatory domain from tightly binding to DgcY.

Specifically, the predicted interaction sites on HhmR are mainly located in three regions, P174–E180, L239–V242, and S258–R260 (Fig. 7F). To validate the role of these regions in the interaction, we performed site-directed mutations at corresponding sites D179, L239, Q240, and G259. MST assays using the purified mutants revealed that the Q240D mutation significantly decreased the binding affinity to DgcY_202–406_ by approximately 11-fold (Fig. 7I, *K_D_* of 570.71 nM). For the remaining mutants (D179A, L239, and G259H), no interaction was observed (Fig. 7G, H, and J). Furthermore, a triple mutant (L239R/Q240D/G259H) also failed to interact with DgcY_202–406_ (Fig. 7K). To rule out the possibility that loss of binding was due to protein instability, we performed the thermal shift assays and compared the melting temperature (*T*_m_) values of these mutants. Except for two L239R-involved mutants that showed a 5°C decrease, D179A, Q240D, and G259H all exhibited constant *T_m_* values as that of HhmR_90–305_ (Fig. S6C). This indicated that the loss or reduction in binding affinity of three mutants is not attributable to protein misfolding or instability. Collectively, these results confirmed that the RD of HhmR serves as the functional binding domain for DgcY_202–406_, and the regions encompassing P174–E180, L239–V242, and S258–R260 are critical for the interaction.

## DISCUSSION

As a halotolerant and alkaliphilic strain, *H. hydrothermalis* Y2 exploits several strategies to maintain osmotic homeostasis in highly saline environments, including the use of various Na^+^/H^+^ transporters and the accumulation of ectoine as a major compatible solute (28). In strain Y2, we have revealed that intracellular ectoine can be metabolized under reduced saline conditions, via the Doe degradative pathway (27). The unique architecture of the *dgcY/doe* hybrid gene cluster implies that the associated c-di-GMP signaling pathway may play a regulatory role in osmotic balance. This hypothesis is supported by multiple lines of experimental evidence. First, disruption of DgcY apparently impaired the growth of strain Y2 under various inorganic salts at low temperatures (15°C and 20°C). Second, elevated NaCl concentrations significantly promoted biofilm formation in strain Y2 carrying pBBR1MCS-5-*dgcY* but not pBBR1MCS-5-*dgcY_202–406_*. This suggests that this TM-fused diguanylate cyclase DgcY plays a role in osmotic regulation and contributes to the halotolerance of *H. hydrothermalis* Y2. Third, this hypothesis is further supported by the potential presence of a similar scaffold of the EX-site within the TM domain of DgcY (Fig. 1E). Combining the diminished Congo red-binding ability of corresponding mutants at the predicted Ex-site, it is reasonable to propose that the relatively conserved acidic residues in this region form a functional EX-site, which may serve as a sensor for ion signals.

Several membrane signal transduction systems, such as two-component systems (41, 42) and mechanosensitive channels (43), have been shown to play critical roles in bacterial osmotic response, ensuring timely activation of adaptive mechanisms of the cells. These modules typically rely on the membrane-associated sensor domain to perceive and transmit the osmotic signals. For instance, the histidine kinase CfcA, which integrates the membrane-anchored CHASE3 and GAF sensor domains, functions as a salt sensor and subsequently regulates the DGC activity of CfcR (44). Our prediction of the salt sensor within DgcY suggests that the TM domains of certain DGCs may likewise serve as a salt signal sensor and contribute to osmotic regulation. Interestingly, lower temperature appears to be essential to the inorganic salt perception by DgcY. We hypothesize that the osmotic regulatory function of DgcY may operate synergistically with temperature. Bacteria have evolved several mechanisms to resist external temperature variations (45), including RNA thermometers that regulate the encoding efficiency of heat shock proteins (46), temperature-sensitive transcription factors (47), and TM proteins capable of sensing changes in lipid bilayer thickness associated with temperature shifts (48, 49). Besides these, DCG-associated modules have also been revealed as thermosensors, such as the GGDEF domain of BtsD (50) and the PAS domain of TdcA (45). As a DGC with functional TM modules, it remains to be determined whether DgcY senses variations in membrane thickness and/or inorganic ions at a lower temperature, undergoes conformational changes, and subsequently triggers the c-di-GMP signaling pathway. In addition, the possibility that a downstream c-di-GMP effector in the DgcY-involved signaling network can respond to temperature cues also needs to be further addressed.

Due to the complexity of c-di-GMP-involved signaling networks, the functional specificity and signaling network of individual DGCs remain a central topic of research. Among the proteins identified in the DgcY_202–406_-binding assay, we are particularly interested in the HhmR regulator, given the similarity between the recorded functions of LTTR family members and the c-di-GMP signaling networks, including bacterial quorum sensing, biofilm formation, virulence, and response to environmental stress (36, 37). Consequently, *in vitro* experiments showed that HhmR modestly affected the DGC activity of DgcY. By contrast, the Y2/Δ*hhmR* mutant showed significant changes in growth rate, motility, and biofilm formation in *H. hydrothermalis* Y2. We propose that the formation of the DgcY/HhmR complex mediates a negative regulation to DgcY activity, which partially accounts for the phenotypic alterations observed upon *hhmR* deletion strain. Beyond this specific interaction, we hypothesize that HhmR may serve as a broader regulatory factor within the c-di-GMP signaling networks in strain Y2. It may potentially modulate the expression of genes related to growth and polysaccharide phenotypes, regulate the transcription of some *dgc* genes, or even form a similar physical interaction with other DGCs, thus resulting in marked phenotypic changes. To the best of our knowledge, literature describing c-di-GMP-related LTTR family regulatory activity is scant. Smitha et al. found that the addition of ppGpp or c-di-GMP attenuates binding of the LTTR family transcriptional regulator XanR to the *xan* operon in *Ralstonia solanacearum* (51). In the YajQ–LysR system identified in *Xanthomonas campestris* and *Lysobacter enzymogenes*, YajQ family protein XC_3703 is a c-di-GMP receptor for downstream regulation (52, 53). Moreover, XC_3703 is found to interact with the LTTR family regulator (XC_2801), and the interaction positively promotes the binding between XC_2801 and promoters of target virulence genes (52). Different from these LTTR regulatory effects and of note, our results verified that HhmR participates in the c-di-GMP signaling pathway, interestingly serving as a DGC partner but not a c-di-GMP effector. Regarding c-di-GMP, it seems to play a novel role in this regulatory network. With weak binding affinity to DgcY_202–406_ and no interaction with HhmR, it does not affect the DGC activity directly, but exerts its inhibitory function by promoting the physical interaction between DgcY and HhmR. We suppose that its binding with DgcY_202–406_ may cause conformational changes of the DGC structure and further intensify the complex formation of HhmR/DgcY, consequently decreasing DGC activity.

**Fig 8:**
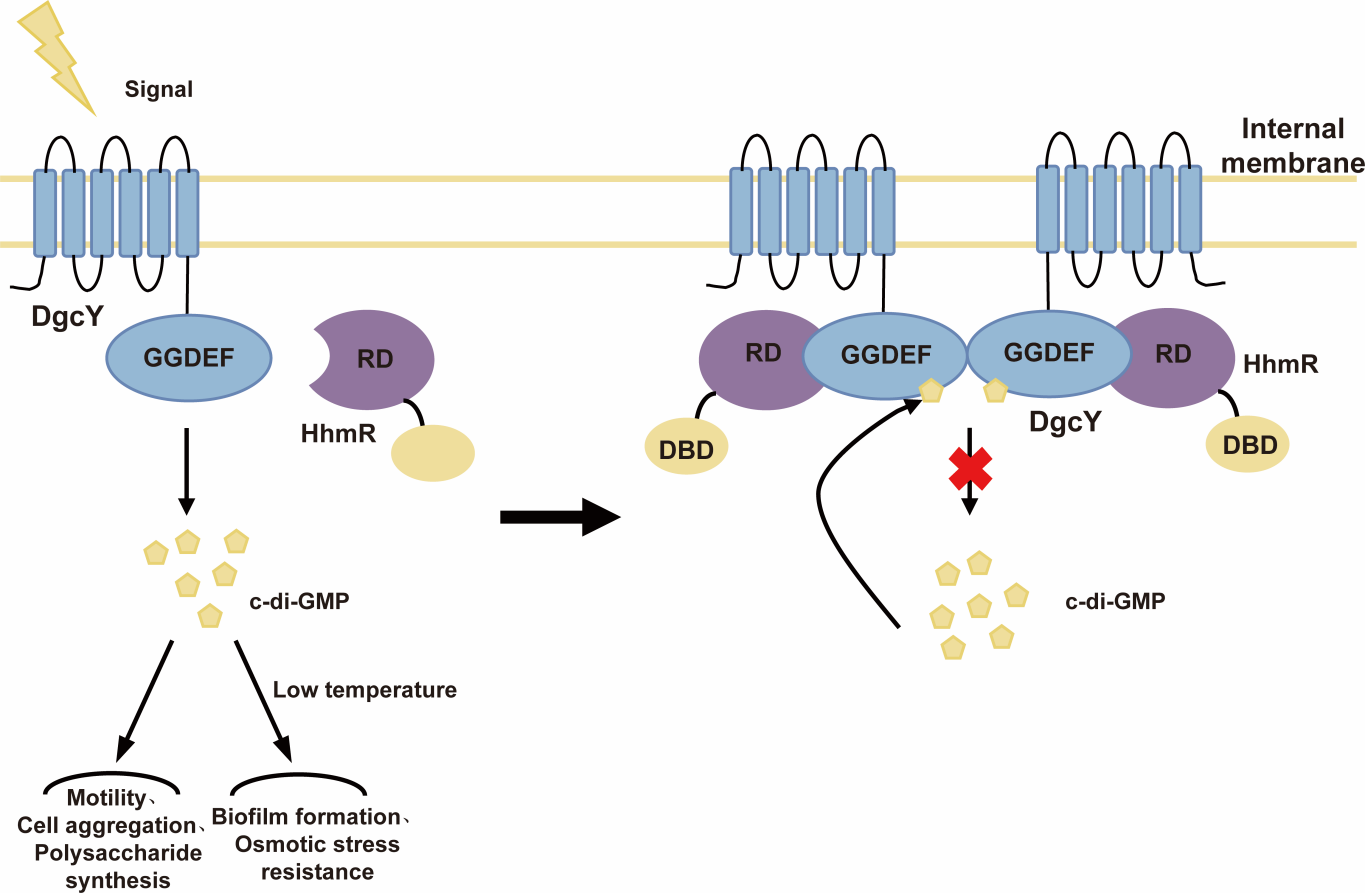
The proposed DgcY-involved signal pathway and regulatory mechanism in *H. hydrothermalis* Y2. As the TM domain of DgcY senses the signal, it transmits the signal to the GGDEF domain via the HD region, promoting active dimer assembly and regulating multiple functions in *H. hydrothermalis* Y2, including swimming motility, cell aggregation, polysaccharide synthesis, and especially biofilm formation and osmotic stress resistance at low temperature. Furthermore, HhmR is recruited and binds to the DgcY active dimer and represses its catalytic activity, reducing c-di-GMP synthesis. c-di-GMP further promotes the complex formation of DgcY-HhmR and strengthens the repression.

In summary, this study elucidates a synergistic regulator mode involved in c-di-GMP and transcriptional regulator in the c-di-GMP signaling pathway of *H. hydrothermalis* Y2 (Fig. 8), specifically as follows: Upon exposure to environmental stimuli, particularly elevated Na^+^ concentrations at low temperatures, the DGC activity of DgcY was activated. The produced c-di-GMP promotes adaptive phenotypes upon the osmotic stress under lower-temperature conditions. As intracellular c-di-GMP accumulates, it facilitates a physical interaction between DgcY_202–406_ and the RD region of HhmR, which subsequently represses the DgcY activity and maintains the intracellular balance.

## MATERIALS AND METHODS

### Culture conditions and strain construction

The bacterial strains and plasmids used in this study are listed in Table S4. Planktonic cultures of *E. coli* strains and *H. hydrothermalis* Y2 strains were grown at 37°C and 30°C, respectively, at 200 rpm in LB medium. For plasmid selection and/or maintenance, antibiotics kanamycin (50 µg mL^−1^), ampicillin (100 µg mL^−1^), and gentamycin (50 µg mL^−1^) were used when necessary.

### Genetic procedures

The *dgcY-* and *hhmR*-related genes were cloned from *H. hydrothermalis* Y2 (accession no. SRP073747). The primers used for construction are listed in Table S5. All constructions were verified by PCR and sequencing.

Conjugative transfer was employed to introduce the plasmids into *H. hydrothermalis* Y2 (54). In brief, the constructed plasmids were first transformed into strain *E. coli* S17-1, followed by mixing the *E. coli* S17-1 derivatives with *H. hydrothermalis* Y2 in the LB plate and co-cultivating at 37°C overnight. Subsequently, the bacterial lawn was resuspended in stroke-physiological saline solution (0.1%, wt/vol) and spread onto an LB plate that was supplemented with ampicillin (100 µg mL^−1^) to select the right strains harboring the constructed plasmid.

For gene knockout, the pK18mob*sacB* system was used. In brief, the upstream and downstream of the knockout gene fragment were amplified, recombined by overlap-extension PCR, and digested using restriction enzymes. The digested recombinant fragment was inserted into the suicide plasmid pK18mob*sacB* at the corresponding restriction sites. The resulting plasmid was transformed into *H. hydrothermalis* Y2 through conjugative transfer as described above. Single crossover cells were selected by LB medium with kanamycin (50 µg mL^−1^) and ampicillin (100 µg mL^−1^) additions. The second crossover cells were isolated on the LB plate containing 20% of sucrose (wt/vol) and finally verified by PCR amplification and DNA sequencing.

QuickChange site-directed mutagenesis was used to introduce specific mutations into the plasmids, using primers listed in Table S5. The PCR product was treated by the *Dpn* I restriction endonuclease at 37°C for 30 min and transformed into *E. coli* DH5α to obtain the site-directed mutagenesis plasmids. The resulting plasmids were isolated and further verified by PCR amplification and DNA sequencing.

### Bioinformative analysis

The protein sequences were submitted to HMMTOP (55) and SMART (56, 57) for topological structure prediction. The three-dimensional structure was predicted by SWISS-MODEL (58, 59) (https://swissmodel.expasy.org/) and AlphaFold3 (60). The sequence was submitted to the AlphaFold3 public web server (https://www.alphafoldserver.com), which generated five models ranked by AlphaFold3 ranking score. The model with the highest confidence score was selected. For evolutionary conservation analysis, multiple sequence alignments were performed by the ClustalW (61) method in MEGA 7.0 (62) software with default parameters. Subsequently, the aligned results were analyzed using ESPript 3.0 (63) (https://espript.ibcp.fr/ESPript/ESPript/) in the EXPert mode with default parameters.

### Protein expression and purification

*E. coli* BL21(DE3) or *E. coli* Rosetta (DE3) constructs carrying the expression plasmids were cultured in LB medium at 37°C until the OD_600_ reached 0.6. Then, isopropyl β-D-1-thiogalactopyranoside (IPTG, final concentration 0.4 mM) was added, and the culture was incubated at 16°C for 12 h. Cells were harvested by centrifugation at 6,000 × *g* and 4°C for 10 min and resuspended in the lysis buffer (150 mM NaCl, 25 mM Tris-HCl, pH 8.0) supplemented with 0.1 mM phenylmethylsulfonyl fluoride (PMSF). The cell resuspension was disrupted with a high-pressure cell disruptor, and debris was removed by centrifuging at 12,000 × *g* for 10 min at 4°C. For His-tag protein purification, the supernatant was loaded onto a Ni-TED NUPharose FF pre-packed column, and the target protein was eluted using elution buffer (150 mM NaCl, 250 mM imidazole, 25 mM Tris-HCl, pH 8.0). For Strep-Tag II protein purification, cells were resuspended in binding buffer (150  mM NaCl, 10  mM Tris–HCl, 1  mM EDTA, pH 8.0), and the target protein was collected as described above. The supernatant was incubated with the Strep-Tag II magnetic beads for 1 h and washed five times with binding buffer. The target protein was eluted using binding buffer containing 2.5  mM of desthiobiotin. Finally, the protein was purified by gel filtration chromatography, using a Superdex 200 column equilibrated with lysis buffer.

### Western blot assay

Proteins were resuspended in SDS loading buffer and separated on a 10% or 15% SDS-PAGE gel. The gel was then transferred to nitrocellulose membranes (Millipore, Germany) and blocked with 3% bovine serum albumin for 1 h. Then, membranes were incubated with the Monoclonal antibody (Abcam), including anti-His and anti-GST Mouse Monoclonal antibody (Proteintech), or anti-Strep-tagII Rabbit, at 1:1000 dilution for 1 h. Subsequently, membranes were washed five times with TBST buffer and incubated with HRP-conjugated Affinipure Goat Anti-Mouse IgG (H + L) or HRP-conjugated Affinipure Goat Anti-Rabbit IgG (H + L) antibody, at a 1:1,000 dilution. Finally, the membranes were washed with TBST buffer and stained using the ECL Detect kit (Proteintech) before imaging on a chemiluminescent imager.

### Congo red binding assay

*E. coli* BL21(DE3) strains that carry the expression plasmids were cultured in the LB medium overnight and then subcultured to an OD_600_ of 0.6. Next, 8 μL of culture was dropped onto the LB agar medium containing Congo red (40 μg mL^−1^), Coomassie brilliant blue (20 μg mL^−1^), and IPTG (0.5 mM). The plates were incubated at 30°C for 2 d before recording (18).

### Aggregation assay

Overnight cultures of the strains were subcultured in fresh LB medium to an OD_600_ of 1. Then, 2 mL of the culture was centrifuged at 6,000 × *g* for 3 min and resuspended in the M9 medium (6.78 g L^−1^ Na_2_HPO_4_, 3 g L^−1^ KH_2_PO_4_, 0.5 g L^−1^ NaCl, 1 g L^−1^ NH_4_Cl, 4 g L^−1^ glucose, 0.241 g L^−1^ MgSO_4_, and 0.011 g L^−1^ CaCl_2_) supplemented with 40 µg mL^−1^ Congo red. The culture was incubated at 30°C for 3 h and centrifuged, allowing adsorbed Congo red to sediment. The absorbance values at 490 nm (A_490_) of supernatant were recorded to quantify the residual Congo red in the medium. In addition, M9 medium containing 40 µg mL^−1^ Congo red was measured and used as a positive control. The amount of Congo red adsorbed by the bacteria was calculated by subtracting the A_490_ value of the supernatant from that of the positive control.

### Biofilm formation assay

Biofilm formation was determined via crystal violet staining as previously described (64). Briefly, the culture was diluted to a final OD_600_ of 0.2 after growing overnight in LB medium and incubated for 24 h to allow biofilm formation. After removing the planktonic cells, every well was washed twice with sterile phosphate-buffered saline (PBS, 8 g L^−1^ NaCl, 0.2 g L^−1^ KCl, 1.44 g L^−1^ Na_2_HPO_4_, 0.24 g L^−1^ KH_2_PO_4_, pH 7.2–7.4), and 200 µL methanol was added for cell fixation. Then, adherent cells were stained with crystal violet (0.1%, wt/vol), and acetic acid (30%, wt/vol) was added to dissolve biofilm-associated crystal violet. The optical density at 570 nm (OD_570_) of the resulting solution was measured to quantify biofilm formation.

### Motility assay

Swimming motility assays were performed as previously described (65). Briefly, overnight cultures were subcultured to an OD_600_ of 1.5 and stabbed into Jensen’s 0.3% agar plate. After incubation at 30°C for 16 h, the swimming diameter was measured and recorded.

### *In vitro* DGC activity assay

With or without 15 µM purified HhmR addition, 10 µM purified DgcY_202–406_ was added to the reaction mixture (50 mM Tris-HCl, 250 mM NaCl, 10 mM MgCl_2_, pH 8.0). The reaction was initiated by adding 200 µM GTP and incubating at 37°C for different times as required. After the reaction, the mixtures were incubated at 100°C for 15 min to stop the reaction, followed by centrifugation at 15,000 × *g* for 10 min. The c-di-GMP concentration was measured by high-performance liquid chromatography (HPLC), which is equipped with a UV detector set to a wavelength of 254 nm. The supernatant was separated by a reverse-phase C_18_ column (250  ×  4.6  mm, Agilent), with a mobile phase comprising 98% buffer A (150 mM Na_2_HPO_4_, pH 5.2) and 2% buffer B (acetonitrile), at a flow rate of 1 mL min^−1^ (25). Commercially available GTP (Sigma, G8877) and c-di-GMP (Sigma, SML1228) were used as standards.

To evaluate the integrated effect of c-di-GMP and HhmR on the DGC activity of DgcY_202–406_, 15 µm c-di-GMP was added to the initiated reaction mixture, and the amount of c-di-GMP at 0 h was recorded as the starting amount of c-di-GMP. The c-di-GMP production was calculated by subtracting the starting amount of c-di-GMP from that detected at 4 h and 8 h.

### Growth of *H. hydrothermalis* Y2 strains

*H. hydrothermalis* Y2 constructs were cultured overnight and subcultured in the LB0 medium, or LB0 medium with different concentrations of NaCl, KCl, MgCl_2_, NaNO_3_, KNO_3_, D-Glucose, D-Sorbitol, and sucrose. With an initial OD_600_ of 0.2, the strains were subcultured at 15°C, 20°C, 25°C, and 30°C for 24  h and sampled for measuring the OD_600_ values.

### GST pull-down assay

The GST pull-down assay was performed using a GST pull-down kit (FITGENE) following the manufacturer’s instructions. Briefly, the GST-tagged fusion protein was expressed and incubated with the Glutathione Agarose resin for 2 h to obtain the GST-tagged fusion protein resin. Then, the mixture was co-incubated with the bacterial lysate at 4°C overnight. After washing to remove unbound proteins, the pull-down products were obtained by vortex shaking and subjected to SDS-PAGE analysis. The resulting gel was sent to Beijing Protein Innovation Co., Ltd. for liquid chromatography-tandem mass spectrometry (LC-MS/MS) analysis. In addition, the pull-down products were further validated by western blot analysis as described above.

### MST assay

The His-tag fusion protein was expressed and purified as described above. The purified protein was diluted to 900 nM using the PBS-T buffer and labeled using the MO His-tag Protein Labeling Kit Red-tris-NTA (Nanotemper Technologies). Ligand stock solution was twofold serial dilutions in the PBS-T buffer and mixed at a 1:1 ratio with 10 µL of the labeled protein solution. All MST measurements were performed at 25°C on the Monolith NT standard capillaries (NanoTemper Technologies) and Monolith device (NanoTemper Technologies) using the Auto-detected power. Binding curves and *K*_D_ values were calculated and fitted by MO Affinity Analysis V10 software (NanoTemper Technologies).

### Docking models for DgcY_202-406_-HhmR based on *in silico* analysis

ZDOCK server (66) (https://zdock.wenglab.org/) was used to predict possible binding modes in the translational and rotational space between DgcY_202-406_ and HhmR based on Fast Fourier Transform. ZDOCK 2.3.2 (67) was used to predict ACE statistical potential, shape complementarity, and electrostatics. The final determined interaction model was validated by PISA (https://www.ebi.ac.uk/pdbe/pisa/).

### Thermal shift assay

Protein was expressed and purified as described above. The purified protein (8 µM) was incubated in reaction buffer (150 mM NaCl, 25 mM Tris-HCl, pH 8.0) at a final volume of 20 µL for 10 min and subsequently loaded into Tycho NT.6 capillaries (Nanotemper Technologies). Then the capillaries were loaded into the Tycho NT.6 instrument (Nanotemper Technologies). Each sample was heated from 35°C to 95°C, and the brightness at 330 and 350 nm against the temperature was detected and measured automatically. *T*_m_ showed the unfolding transition in the 350/330 nm ratio signal.

### Statistical analysis and reproducibility

All experiments were repeated at least three times. GraphPad Prism version 8.0.2 was used for statistical analyses, including T-tests, one-Way ANOVA, and two-Way ANOVA. Values of *P* < 0.05 were considered statistically significant.

## SUPPLEMENTAL MATERIAL

Supplemental figures and tables mbio.01498-25-s0001.docx Fig. S1 to S6; Tables S1 to S5.

ASM does not own the copyrights to Supplemental Material that may be linked to, or accessed through, an article. The authors have granted ASM a non-exclusive, world-wide license to publish the Supplemental Material files. Please contact the corresponding author directly for reuse.
