# Supplementary material for: Dual regulatory role of cyclic di-GMP via the DgcY/HhmR complex in salt response of Halomonas hydrothermalis Y2
Source: mBio. 2025 Aug 6;16(9):e01498-25. doi: 10.1128/mbio.01498-25 (PMC12421958; doi:10.1128/mbio.01498-25)
Supplement: Supplemental figures and tables — Fig. S1 to S6; Tables S1 to S5. [file mbio.01498-25-s0001.docx]

**Dual regulatory role of cyclic di-GMP via the DgcY/HhmR complex in salt response of *Halomonas hydrothermalis* Y2**

Ye Zhang^1^, Wenkai Wang^1^, Yuanxiang Liu^1^, Chongzhou Li^1^, Jinyan Liu^1^, Luhua Feng^1^, Yan Gao^1^, Yishu Peng^1^, Wei Wang^1^, Chunfang Li^1^, Ping Xu^2^, Chunyu Yang^1^*

1. State Key Laboratory of Microbial Technology, Institute of Microbial Technology,

Shandong University, Qingdao, 266237, People's Republic of China

1. State Key Laboratory of Microbial Metabolism, Shanghai Jiao Tong University, People's Republic of China

*Corresponding author: Prof. Chunyu Yang

E-mail: [ycy21th@sdu.edu.cn](mailto:ycy21th@sdu.edu.cn)

Telephone: 86-0532-58631501


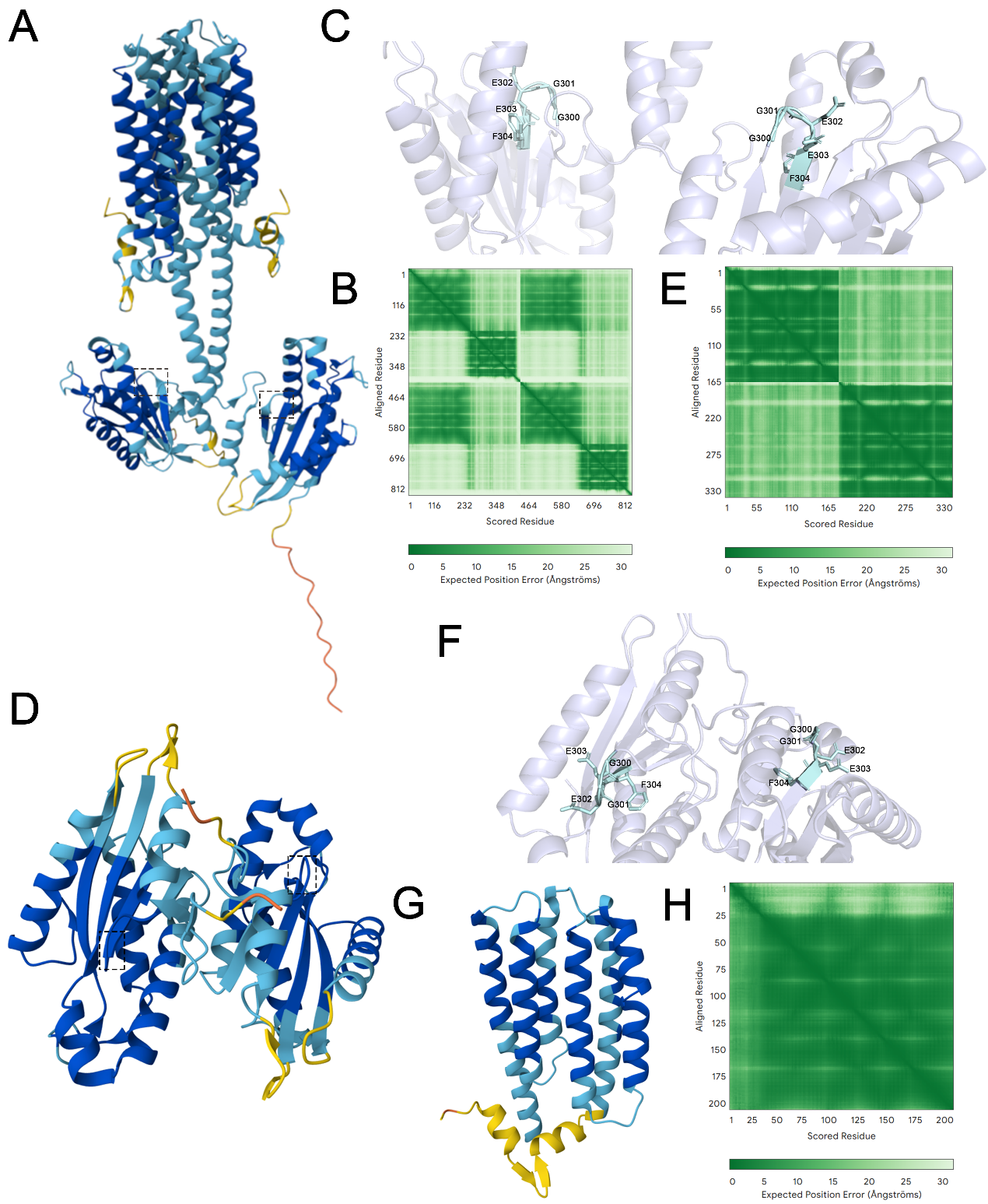


**Supplementary Fig. 1 |** **Predicted structure of DgcY fragments by AlphaFold3. A.** Predicted dimer structure of DgcY by AlphaFold3. The structure was colored according to the confidence level of AlphaFold3. Regions with high Predicted Local Distance Difference Test (plDDT) values (> 90) are colored in dark blue, 70 < pLDDT ≤ 90 are colored light blue, 50 < pLDDT ≤ 70 are colored in yellow, pLDDT ≤ 50 are colored in orange. The interface predicted TM-score (ipTM) value was 0.6 and predicted TM-score (pTM) value was 0.61. The black dotted box showed the GGEEF domain. **B.** PAE (Predicted Aligned Error) plot of predicted dimer structure of DgcY. **C.** Another view of GGEEF domain in the predicted dimer structure of DgcY. The residues of GGEEF domain were allocated successively with the single-letter identifier of certain amino acid and its corresponding residue number. **D.** Predicted dimer structure of DgcY_219–383_. The ipTM value was 0.42 and pTM value was 0.65. The black dotted box showed the GGEEF domain. **E.** PAE plot of predicted dimer structure of DgcY_219–383_. **F.** Another view of GGEEF domain in the predicted dimer structure of DgcY_219–383_. **G.** Predicted structure of DgcY_1–201_ monomer. The pTM value was 0.82. **H.** PAE plot of predicted structure of DgcY_1–201_ monomer.


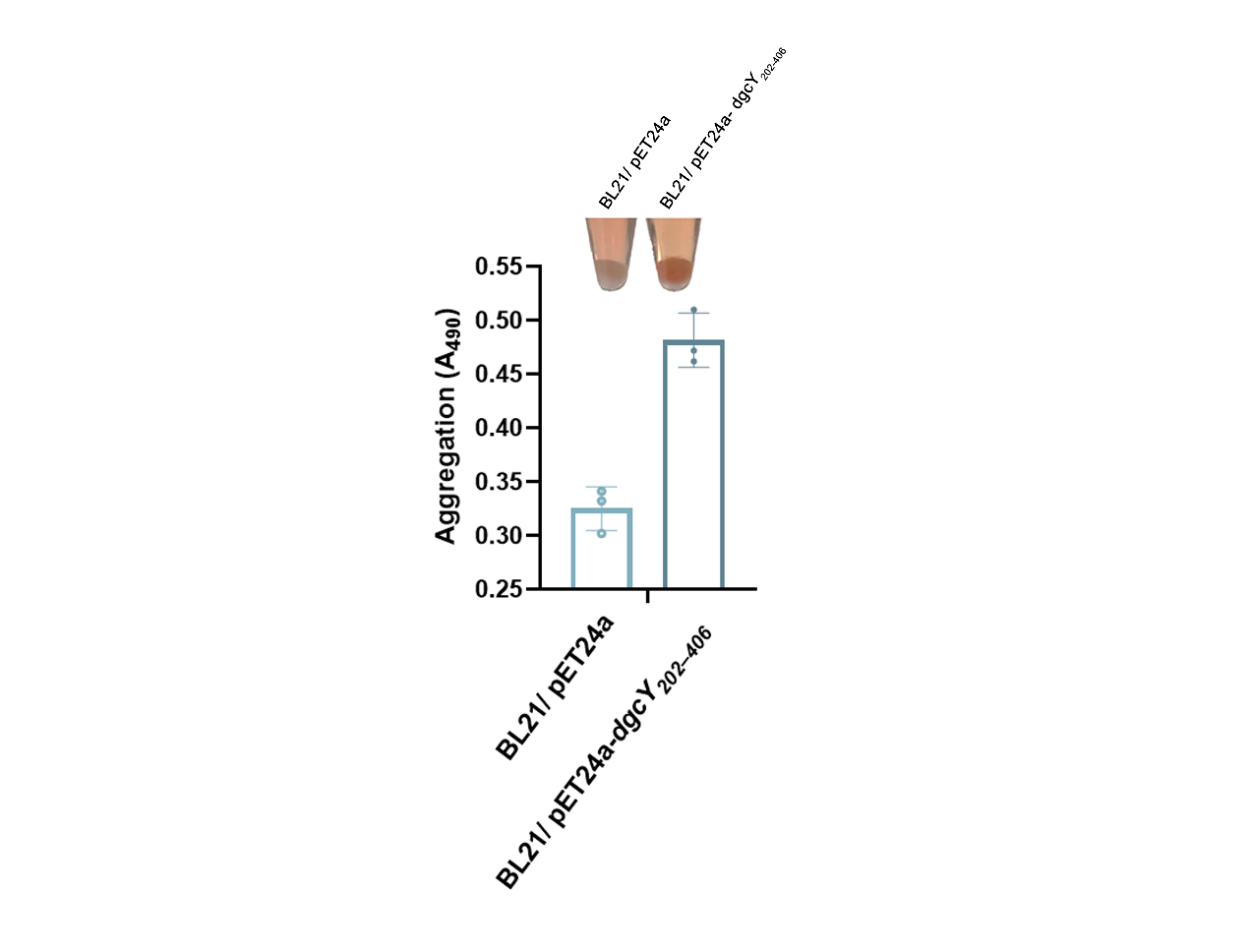


**Supplementary Fig. 2 |** **Cell aggregations of BL21/pET24a and BL21/pET24a-*dgcY_202–406_* in M9 medium supplemented with Congo red.**


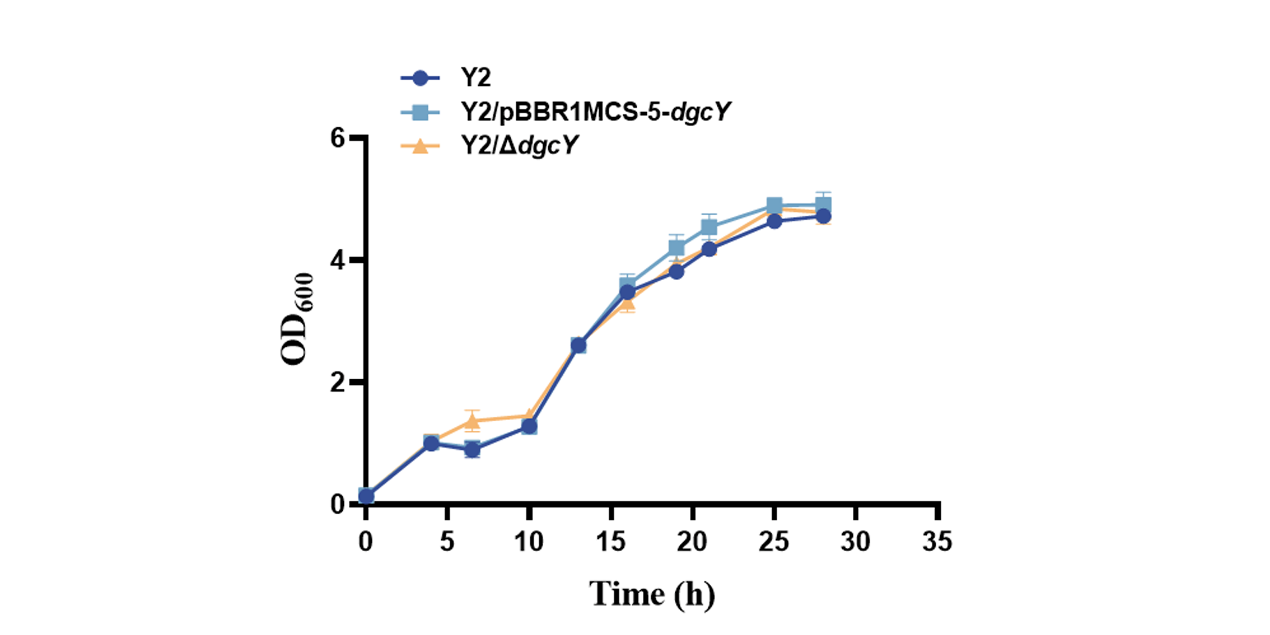
**Supplementary Fig. 3 |** **Growth curves of *H. hydrothermalis* Y2, Y2/pBBR1MCS-5-*dgcY*, and Y2/Δ*dgcY* in LB medium.** The cultures were incubated at 30 °C and 200 rpm.


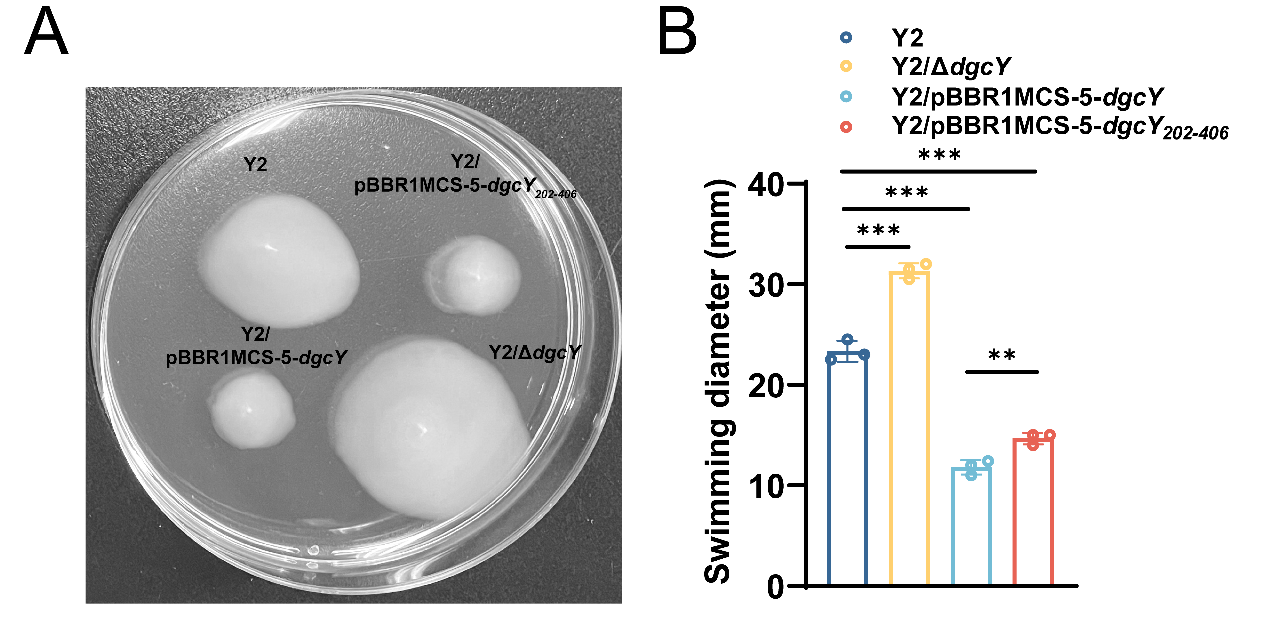


**Supplementary Fig. 4 | Physical functions of the TM domain of DgcY in *H. hydrothermalis* Y2. A-B.** Swimming motility of Y2, Y2/pBBR1MCS-5-*dgcY*, Y2/pBBR1MCS-5-*dgcY_202–406_*, and Y2/Δ*dgcY* in 0.3% soft agar LB plates.


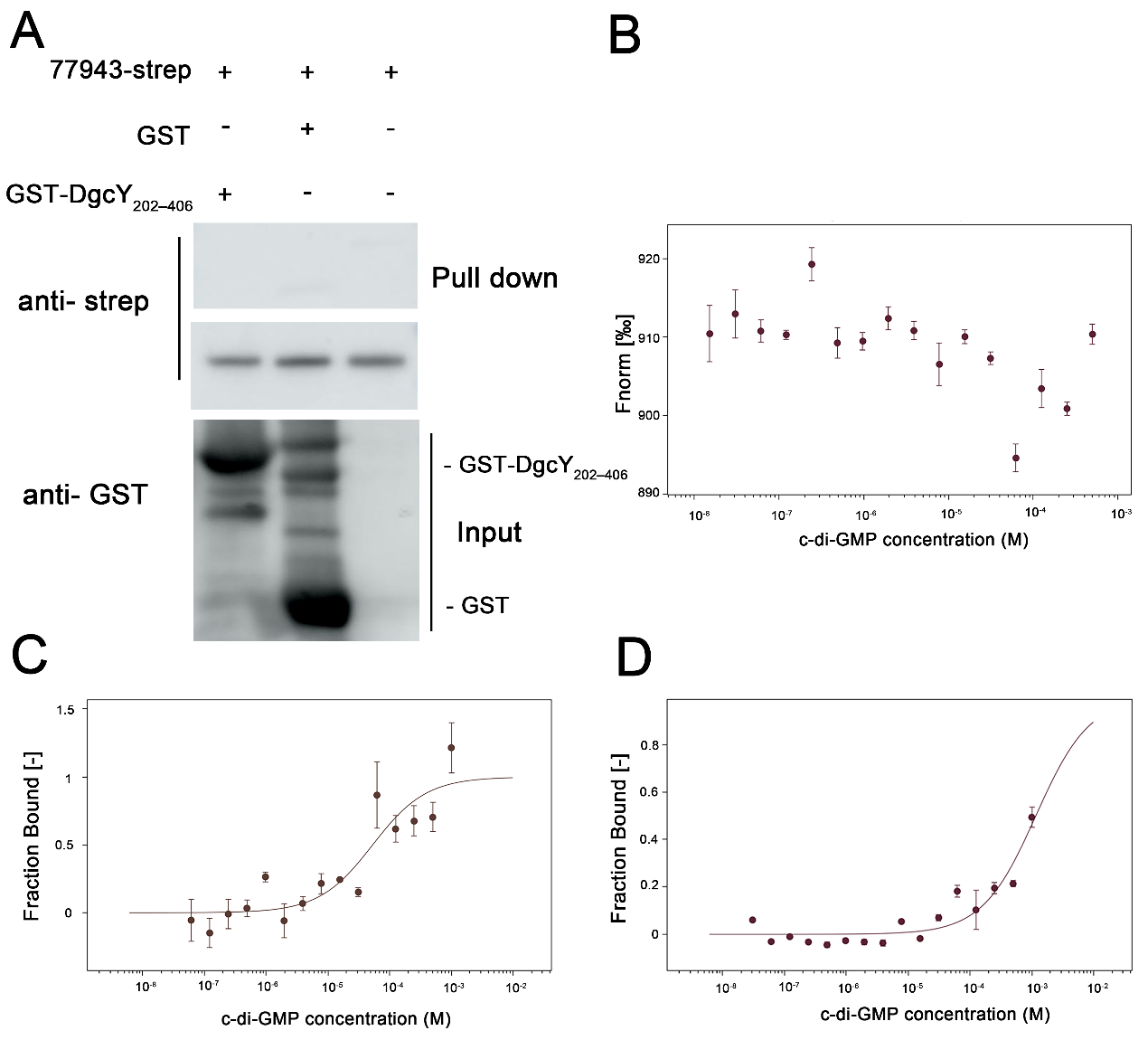


**Supplementary Fig. 5 | The specific interaction between** **DgcY_202_****_–406_ and HhmR. A.** GST pull-down showed that DgcY_202–406_ does not interact with the HhmR homologue 77943. 77943-strep was incubated with GST or GST–DgcY_202__–406_ fused resins, and the interaction complexes were eluted for western blotting. **B.** MST binding curve showing no interaction between HhmR and c-di-GMP. **C.** MST binding curve showing the interaction between DgcY_202–406_ and c-di-GMP, with a *K_D_* value of 53.25 μM. **D.** MST binding curve showing the interaction between DgcY_222–406_ and c-di-GMP, with a *K_D_* value of 1.17 mM.


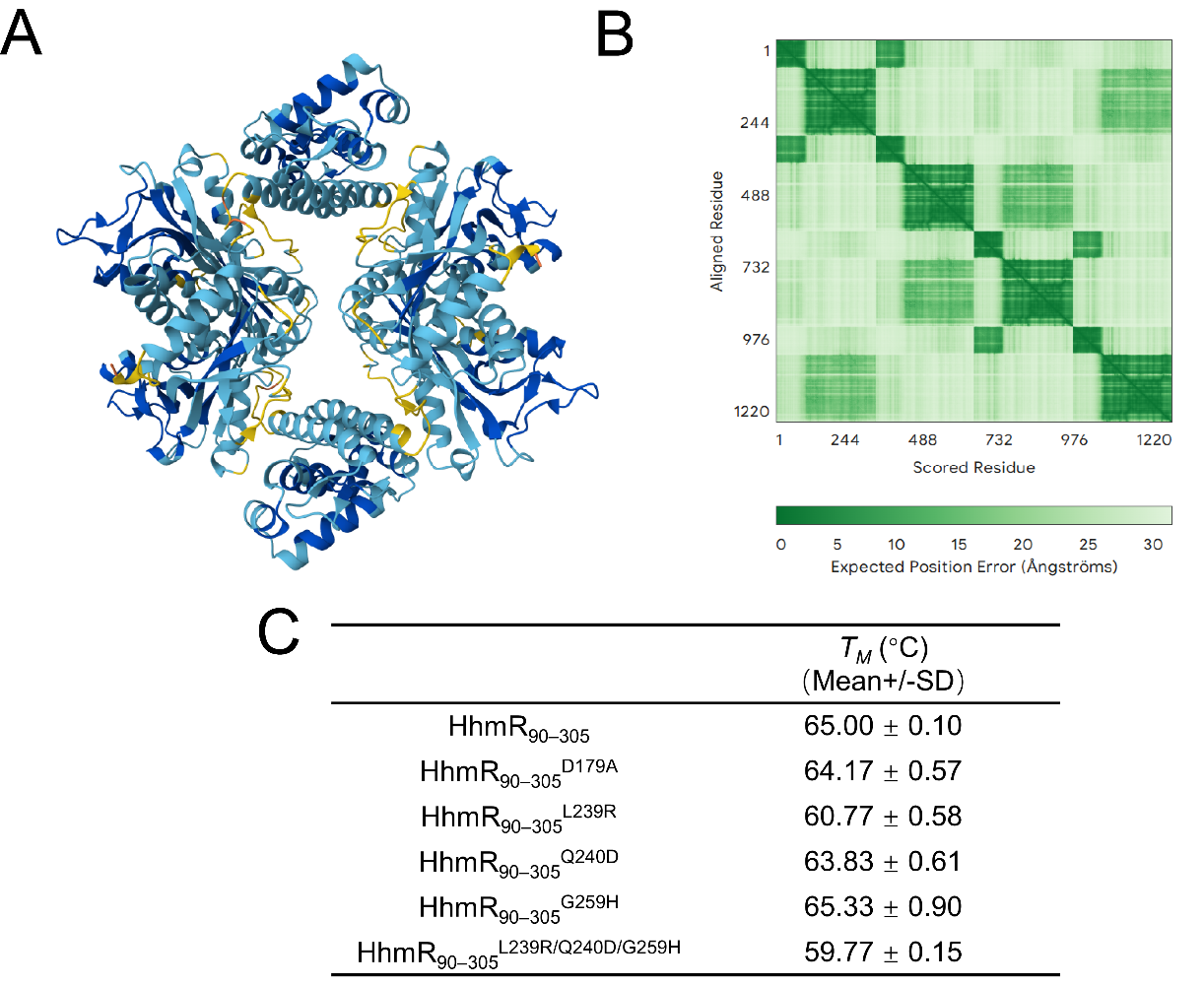
**Supplementary Fig. 6 |** **Predicted tetrameric structure of HhmR by AlphaFold3 and the *T_m_* values of HhmR_90-305_ mutants. A.** Predicted tetrameric structure of HhmR by AlphaFold3. The structure was colored according to the confidence level of AlphaFold3. The ipTM value was 0.3 and pTM value was 0.4. **B.** PAE plot of tetrameric structure of HhmR. **C.** *T_m_* values of HhmR_90-305_ mutants.

**Table S1** Q Exactive LC/MS analyse for GST pull-down elution of GST- DgcY_202–406_ with the culture lysis of *H. hydrothermalis* Y2

| Accession | Description | Mass | Score | Matches | Sequences | emPAI | Coverage |
| --- | --- | --- | --- | --- | --- | --- | --- |
| gi\|1250063719 | GGDEF domain-containing protein [*H. hydrothermalis*] | 45414 | 7529 | 239(200) | 11(11) | 5.20 | 35% |
| gi\|730431947 | acyl-CoA dehydrogenase [*H. hydrothermalis*] | 89839 | 396 | 36(29) | 2(1) | 0.04 | 1% |
| gi\|730430886 | elongation factor Tu [*H. hydrothermalis*] | 43632 | 321 | 20(17) | 12(11) | 1.78 | 42% |
| gi\|730430394 | 2,4-dienoyl-CoA reductase [*H. hydrothermalis*] | 72988 | 147 | 16(16) | 1(1) | 0.04 | 0% |
| gi\|730431835 | LysR family transcriptional regulator [*H. hydrothermalis*] | 34447 | 102 | 12(6) | 1(1) | 0.10 | 2% |
| gi\|1820235774 | potassium channel protein [*H. hydrothermalis*] | 37923 | 92 | 4(3) | 2(1) | 0.09 | 4% |
| gi\|515481263 | MULTISPECIES: succinate dehydrogenase flavoprotein subunit [*Halomonas*] | 64732 | 66 | 1(1) | 1(1) | 0.05 | 2% |
| gi\|730431690 | glycosyl transferase [*H. hydrothermalis*] | 39975 | 57 | 5(4) | 1(1) | 0.08 | 2% |
| gi\|515482920 | MULTISPECIES: cytochrome-c oxidase, cbb3-type subunit II [*Halomonas*] | 22999 | 48 | 4(4) | 1(1) | 0.15 | 3% |
| gi\|730430712 | FAD-linked oxidase [*H. hydrothermalis*] | 114733 | 44 | 11(7) | 1(1) | 0.03 | 0% |
| gi\|1820236630 | copper-translocating P-type ATPase [*H. hydrothermalis*] | 75993 | 35 | 11(5) | 1(1) | 0.04 | 0% |
| gi\|730432378 | 2-oxoglutarate dehydrogenase [*H. hydrothermalis*] | 54661 | 31 | 1(1) | 1(1) | 0.06 | 1% |
| gi\|1957457465 | diguanylate cyclase [*H. hydrothermalis*] | 42390 | 30 | 1(1) | 1(1) | 0.08 | 1% |
| gi\|917627422 | WecB/TagA/CpsF family glycosyltransferase [*H. hydrothermalis*] | 25702 | 29 | 6(1) | 2(1) | 0.13 | 5% |
| gi\|730431293 | glycosyl hydrolase family 32 [*H. hydrothermalis*] | 55698 | 25 | 5(3) | 1(1) | 0.06 | 1% |
| gi\|730432788 | diguanylate phosphodiesterase [*H. hydrothermalis*] | 25930 | 23 | 9(1) | 1(1) | 0.13 | 4% |
| gi\|1820237310 | class I SAM-dependent DNA methyltransferase [*H. hydrothermalis*] | 141439 | 21 | 4(1) | 2(1) | 0.02 | 1% |
| gi\|1250065147 | hypothetical protein CLM76_09640 [*H. hydrothermalis*] | 47005 | 20 | 6(1) | 3(1) | 0.07 | 6% |
| gi\|730432036 | enoyl-CoA hydratase [*H. hydrothermalis*] | 41352 | 20 | 1(1) | 1(1) | 0.08 | 1% |
| gi\|515480447 | MULTISPECIES: DNA-binding transcriptional regulator Fis [*Halomonas*] | 11663 | 19 | 7(2) | 1(1) | 0.30 | 6% |
| gi\|1250066857 | transposase [*H. hydrothermalis*] | 42608 | 17 | 1(0) | 1(0) | 0.08 | 1% |

**Table S2** Q Exactive LC/MS analyse for GST pull-down elution of GST with Y2 lysis

| Accession | Description | Mass | Score | Matches | Sequences | emPAI | Coverage |
| --- | --- | --- | --- | --- | --- | --- | --- |
| gi\|730432719 | hypothetical protein PZ78_02405 [*H. hydrothermalis*] | 45383 | 337 | 20(13) | 6(4) | 0.75 | 12% |
| gi\|730430665 | molecular chaperone DnaK [*H. hydrothermalis*] | 69345 | 296 | 13(8) | 7(5) | 0.32 | 13% |
| gi\|730430886 | elongation factor Tu [*H. hydrothermalis*] | 43632 | 172 | 13(9) | 10(7) | 0.67 | 26% |
| gi\|730431644 | ATP F0F1 synthase subunit alpha [*H. hydrothermalis*] | 56009 | 99 | 6(4) | 3(2) | 0.19 | 7% |
| gi\|515481263 | MULTISPECIES: succinate dehydrogenase flavoprotein subunit [*Halomonas*] | 64732 | 81 | 2(1) | 2(1) | 0.05 | 4% |
| gi\|515482309 | MULTISPECIES: F0F1 ATP synthase subunit beta [*Halomonas*] | 49799 | 80 | 2(1) | 2(1) | 0.07 | 5% |
| gi\|730430494 | Clp protease ATP-binding protein [*H. hydrothermalis*] | 46889 | 76 | 2(2) | 2(2) | 0.15 | 6% |
| gi\|730432681 | aldehyde dehydrogenase [*H. hydrothermalis*] | 55464 | 71 | 2(1) | 1(1) | 0.12 | 2% |
| gi\|1820236961 | aconitate hydratase B [*H. hydrothermalis*] | 94944 | 66 | 4(2) | 3(2) | 0.07 | 4% |
| gi\|730430890 | DNA-directed RNA polymerase subunit beta' [*H. hydrothermalis*] | 155705 | 63 | 21(1) | 4(1) | 0.02 | 3% |
| gi\|515482498 | MULTISPECIES: 50S ribosomal protein L1 [*Halomonas*] | 24380 | 54 | 1(1) | 1(1) | 0.14 | 5% |
| gi\|497409683 | MULTISPECIES: DNA-directed RNA polymerase subunit alpha [*Halomonas*] | 36600 | 46 | 2(1) | 2(1) | 0.09 | 7% |
| gi\|730430234 | FAD synthetase [*H. hydrothermalis*] | 37940 | 42 | 4(3) | 3(2) | 0.18 | 11% |
| gi\|515480434 | MULTISPECIES: chaperonin GroEL [*Halomonas*] | 57817 | 33 | 4(2) | 3(2) | 0.12 | 7% |
| gi\|730432086 | ribonucleotide-diphosphate reductase subunit beta [*H. hydrothermalis*] | 37133 | 26 | 2(1) | 1(1) | 0.09 | 2% |
| gi\|730430070 | response regulator receiver protein [*H. hydrothermalis*] | 44078 | 26 | 2(1) | 1(1) | 0.07 | 1% |
| gi\|497409686 | MULTISPECIES: 30S ribosomal protein S13 [*Halomonas*] | 13327 | 24 | 1(1) | 1(1) | 0.26 | 6% |
| gi\|730432270 | capsule biosynthesis protein [*H. hydrothermalis*] | 75754 | 24 | 2(1) | 1(1) | 0.04 | 1% |
| gi\|730430927 | histidine kinase [*H. hydrothermalis*] | 74561 | 23 | 1(1) | 1(1) | 0.04 | 1% |
| gi\|730432535 | response regulator receiver protein [*H. hydrothermalis*] | 47362 | 23 | 3(1) | 1(1) | 0.07 | 2% |
| gi\|730432943 | DNA polymerase III subunit beta [*H. hydrothermalis*] | 11132 | 19 | 1(1) | 1(1) | 0.31 | 7% |

**Table S3** Q Exactive LC/MS analyse for GST pull-down elution of GST- DgcY_202–406_ with Rosetta/pET24a-*hhmR-strep* lysis

| **Accession** | **Description** | **Mass** | **Score** | **Matches** | **Sequences** | **emPAI** | **Coverage** |
| --- | --- | --- | --- | --- | --- | --- | --- |
| gi\|1250063719 | GGDEF domain-containing protein [*H. hydrothermalis*] | 45414 | 27747 | 674(620) | 17(16) | 19.46 | 46% |
| gi\|1250066203 | LysR family transcriptional regulator [*H. hydrothermalis*] | 34428 | 697 | 25(19) | 12(9) | 2.02 | 60% |
| gi\|730430886 | elongation factor Tu [*H. hydrothermalis*] | 43632 | 138 | 6(6) | 2(2) | 0.44 | 6% |
| gi\|515482309 | MULTISPECIES: F0F1 ATP synthase subunit beta [*Halomonas*] | 49799 | 122 | 4(2) | 3(2) | 0.14 | 9% |
| gi\|1538840490\| | MULTISPECIES: succinate dehydrogenase flavoprotein subunit [*Halomonas*] | 64774 | 112 | 4(1) | 3(1) | 0.05 | 8% |
| gi\|730431883 | 30S ribosomal protein S1 [*H. hydrothermalis*] | 61665 | 81 | 7(3) | 4(2) | 0.17 | 9% |
| gi\|1820235449 | DNA-directed RNA polymerase subunit beta [*H. hydrothermalis*] | 152156 | 77 | 3(2) | 3(2) | 0.04 | 2% |
| gi\|515482521 | MULTISPECIES: 30S ribosomal protein S4 [*Halomonas*] | 23433 | 54 | 8(1) | 4(1) | 0.14 | 16% |
| gi\|2157378130\| | MULTISPECIES: EAL domain-containing protein [*Halomonas*] | 95905 | 53 | 18(6) | 2(1) | 0.03 | 1% |
| gi\|1538838965 | MULTISPECIES: 50S ribosomal protein L17 [*Halomonas*] | 14635 | 52 | 4(1) | 2(1) | 0.23 | 11% |
| gi\|497409698 | MULTISPECIES: 50S ribosomal protein L14 [*Halomonas*] | 13630 | 47 | 1(1) | 1(1) | 0.25 | 5% |
| gi\|730432412 | ABC transporter [*H. hydrothermalis*] | 42263 | 43 | 1(1) | 1(1) | 0.08 | 2% |
| gi\|730431914 | acetyl-CoA carboxylase [*H. hydrothermalis*] | 49055 | 33 | 2(1) | 2(1) | 0.07 | 3% |
| gi\|515481013 | MULTISPECIES: universal stress protein [*Halomonas*] | 16268 | 33 | 1(1) | 1(1) | 0.21 | 4% |
| gi\|1250066661 | response regulator [*H. hydrothermalis*] | 44037 | 32 | 5(3) | 2(1) | 0.07 | 4% |
| gi\|497409683 | MULTISPECIES: DNA-directed RNA polymerase subunit alpha [*Halomonas*] | 36600 | 31 | 1(1) | 1(1) | 0.09 | 2% |
| gi\|730430883 | 50S ribosomal protein L4 [*H. hydrothermalis*] | 22063 | 28 | 1(1) | 1(1) | 0.15 | 3% |
| gi\|496389993 | MULTISPECIES: 30S ribosomal protein S10 [*Halomonas*] | 11818 | 27 | 1(1) | 1(1) | 0.29 | 9% |
| gi\|730432086 | ribonucleotide-diphosphate reductase subunit beta [*H. hydrothermalis*] | 37133 | 24 | 6(1) | 2(1) | 0.09 | 4% |
| gi\|1820236278 | transcriptional regulator [*H. hydrothermalis*] | 37315 | 22 | 5(1) | 1(1) | 0.09 | 2% |
| gi\|515481762 | MULTISPECIES: transcription termination factor Rho [*Halomonas*] | 47160 | 20 | 3(1) | 3(1) | 0.07 | 5% |
| gi\|730431798 | integrase [*H. hydrothermalis*] | 46824 | 20 | 1(1) | 1(1) | 0.07 | 5% |
| gi\|1820239000 | two-component sensor histidine kinase [*H. hydrothermalis*] | 52208 | 16 | 6(1) | 2(1) | 0.06 | 2% |
| gi\|1250066841 | transcriptional regulator [*H. hydrothermalis*] | 17767 | 16 | 13(2) | 1(1) | 0.19 | 5% |
| gi\|496389973 | MULTISPECIES: 50S ribosomal protein L11 [*Halomonas*] | 15010 | 15 | 1(1) | 1(1) | 0.23 | 6% |
| gi\|1820235635 | hypothetical protein HHSLTHF2_03520 [*H. hydrothermalis*] | 91739 | 14 | 4(1) | 2(1) | 0.04 | 1% |

**Table S4** Strains and plasmids used in this study

| **Strains and plasmids** | **Description** |
| --- | --- |
| **Strain** |  |
| *Escherichia coli* BL21(DE3) | F^-^ *ompT* *gal* dcm on *hsdSB*(r_B_^-^ m_B_^-^) λ(DE3 [*lac*I *lac*UV5-T7 *gene* 1 *ind1 sam7 nin5*]) |
| BL21(DE3)/pET24a | *E. coli* BL21(DE3) harboring the empty expression plasmid pET24a |
| BL21(DE3)/pET24a*- dgcY* | *E. coli* BL21(DE3) harboring the expression plasmid pET24a*- dgcY* |
| BL21(DE3)/pET24a-*dgcY_202_****_–_****_406_* | *E. coli* BL21(DE3) harboring the expression plasmid pET24a-*dgcY_202_****_–_****_406_* |
| BL21(DE3)/pET24a-*dgcY_222_****_–_****_406_* | *E. coli BL21(DE3) harboring the expression plasmid pET24a-dgcY_222_****_–_****_406_* |
| BL21(DE3)/pET24a-*gst* | *E. coli BL21(DE3) harboring the expression plasmid pET24a-gst* |
| BL21(DE3)/pET24a-*gst-dgcY_202_****_–_****_406_* | *E. coli* BL21(DE3) harboring the expression plasmid pET24a-*gst-dgcY_202_****_–_****_406_* |
| *E. coli* Rosetta (DE3) | F^-^ *ompT gal dcm on hsdSB(r_B_^-^ m_B_^-^)* λ(DE3)  *pRARE(argU,argW,ilex,glyT,leuW,proL)(Cam^r^)* |
| Rosetta (DE3)/pET24a-*hhmR-strep* | *E. coli* Rosetta (DE3) harboring the expression plasmid pET24a-*hhmR-strep* |
| Rosetta (DE3)/pET24a-*hhmR_1_****_–_****_89_-strep* | *E. coli* Rosetta (DE3) harboring the expression plasmid pET24a*-hhmR_1_****_–_****_89_-strep* |
| Rosetta (DE3)/pET24a*-hhmR_90_****_–_****_305_-strep* | *E. coli* Rosetta (DE3) harboring the expression plasmid pET24a*-hhmR_90_****_–_****_305_-strep* |
| Rosetta (DE3)/pET24a*-hhmR_90_****_–_****_305_^D179A^-strep* | *E. coli* Rosetta (DE3) harboring the expression plasmid pET24a*-hhmR_90_****_–_****_305_^D179A^-strep* |
| Rosetta (DE3)/pET24a*-hhmR_90_****_–_****_305_^L239R^-strep* | *E. coli* Rosetta (DE3) harboring the expression plasmid pET24a*-hhmR_90_****_–_****_305_^L239R^-strep* |
| Rosetta (DE3)/pET24a*-hhmR_90_****_–_****_305_^Q240D^-strep* | *E. coli* Rosetta (DE3) harboring the expression plasmid pET24a*-hhmR_90_****_–_****_305_^Q240D^-strep* |
| Rosetta (DE3)/pET24a*-hhmR_90_****_–_****_305_^G259H^-strep* | *E. coli* Rosetta (DE3) harboring the expression plasmid pET24a*-hhmR_90_****_–_****_305_ ^G259H^ -strep* |
| Rosetta (DE3)/pET24a*-hhmR_90_****_–_****_305_^L239R/Q240D/G259H^-strep* | *E. coli* Rosetta (DE3) harboring the expression plasmid pET24a*-hhmR_90_****_–_****_305_^L239R/Q240D/G259H^-strep* |
| *E. coli* DH5α | F^-^ φ80d lacZΔM15 Δ(*lacZYA*-*argF*) U169 *endA*1 *recA*1 *hsdR*17(r_k_^-^ m_k_^+^) *supE*44λ^-^ *thi*-1 *gyrA*96 *relA*1 *phoA* |
| *E. coli* S17-1 | *RP4-2(Km::Tn7,Tc::Mu-1) pro-82 LAMpir, recA1 endA1 thiE1 hsdR17 creC510* |
| *E. coli*S17-1 pK18mob*sacB*-*ΔdgcY* | *E. coli*S17-1 harboring the suicide plasmid pK18mob*sacB*-*ΔdgcY* |
| *E. coli*S17-1 pK18mob*sacB*-*ΔhhmR* | *E. coli*S17-1 harboring the suicide plasmid pK18mob*sacB*-*ΔhhmR* |
| *E. coli*S17-1 pBBR1 mcs5-*dgcY* | *E. coli*S17-1 harboring the broad-host-range plasmid pBBR1 mcs5-*dgcY* |
| *E. coli*S17-1 pBBR1 mcs5-*dgcY_202_****_–_****_406_* | *E. coli*S17-1 harboring the broad-host-range plasmid pBBR1 mcs5-*dgcY_202_****_–_****_406_* |
| *E. coli* S17-1 pBBR1 mcs5*-hhmR* | *E. coli*S17-1 harboring the broad-host-range plasmid pBBR1 mcs5-*hhmR* |
| *H. hydrothermalis* Y2 | Wild-type |
| *H. hydrothermalis* Y2/*ΔdgcY* | *H. hydrothermalis* Y2 with a deletion of the *dgcY* gene |
| *H. hydrothermalis* Y2/Δ*hhmR* | *H. hydrothermalis* Y2 with a deletion of the *hhmR* gene |
| *H. hydrothermalis* Y2/pBBR1 mcs5- *dgcY* | *H. hydrothermalis* Y2 harboring the plasmid pBBR1 mcs5- *dgcY* |
| *H. hydrothermalis* Y2/pBBR1 mcs5- *dgcY_202_****_–_****_406_* | *H. hydrothermalis* Y2 harboring the plasmid pBBR1 mcs5- *dgcY_202_****_–_****_406_* |
| *H. hydrothermalis Y2* Δ*hhmR/*pBBR1 mcs5*-hhmR* | *H. hydrothermalis* Y2 with a deletion of the *hhmR* gene broad-host-range plasmid pBBR1 mcs5-*hhmR* |
| **plasmid** |  |
| pET-24a (+) | Vector for protein expression; Km^r^ |
| pET24a-*dgcY* | pET-24a(+) contained *dgcY* gene of *H. hydrothermalis* Y2 |
| pET24a-*dgcY_202_****_–_****_406_* | pET-24a(+) contained *dgcY_202_****_–_****_406_* gene of *H. hydrothermalis* Y2 |
| pET24a-*dgcY_222_****_–_****_406_* | pET-24a(+) contained *dgcY_222_****_–_****_406_* gene of *H. hydrothermalis* Y2 |
| pET24a-*gst* | pET-24a(+) contained *gst* gene |
| pET24a-*gst-dgcY_202_****_–_****_406_* | pET-24a(+) contained *dgcY_202_****_–_****_406_* gene of *H. hydrothermalis* Y2 fused with *gst-tag* gene |
| pET24a-*hhmR-strep* | pET-24a(+) contained *hhmR* gene of *H. hydrothermalis* Y2 fused with *Strep-*tagⅡ gene |
| pET24a-*hhmR_1_****_–_****_89_-strep* | pET-24a(+) contained *hhmR_1_****_–_****_89_* gene of *H. hydrothermalis* Y2 fused with *Strep-*tagⅡ gene |
| pET24a-*hhmR_90_****_–_****_305_-strep* | pET-24a(+) contained *hhmR_90_****_–_****_305_* gene of *H. hydrothermalis* Y2 fused with *Strep-*tagⅡ gene |
| pET24a*-hhmR_90_****_–_****_305_^D179A^-strep* | pET-24a(+) contained *hhmR_90_****_–_****_305_* gene with D179A mutation of *H. hydrothermalis* Y2 fused with *Strep-*tagⅡ gene |
| pET24a*-hhmR_90_****_–_****_305_^L239R^-strep* | pET-24a(+) contained *hhmR_90_****_–_****_305_* gene with L239R mutation of *H. hydrothermalis* Y2 fused with *Strep-*tagⅡ gene |
| pET24a*-hhmR_90_****_–_****_305_^Q240D^-strep* | pET-24a(+) contained *hhmR_90_****_–_****_305_* gene with Q240D mutation of *H. hydrothermalis* Y2 fused with *Strep-*tagⅡ gene |
| pET24a*-hhmR_90_****_–_****_305_^G259H^-strep* | pET-24a(+) contained *hhmR_90_****_–_****_305_* gene with G259H mutation of *H. hydrothermalis* Y2 fused with *Strep-*tagⅡ gene |
| pET24a*-hhmR_90_****_–_****_305_^L239R/Q240D/G259H^-strep* | pET-24a(+) contained *hhmR_90_****_–_****_305_* gene with L239R/Q240D/G259H triple mutation of *H. hydrothermalis* Y2 fused with *Strep-*tagⅡ gene |
| pK18mob*sacB* | Suicide plasmid for gene knockout; Kmr |
| pK18mobsacB-*ΔdgcY* | Partial lengths of *dgcY* were inserted into pK18mobsacB |
| pK18mobsacB-*ΔhhmR* | Partial lengths of *hhmR* were inserted into pK18mobsacB |
| pBBR1MCS-5 | broad-host-range (bhr) plasmid; Gm^r^ |
| pBBR1MCS-5-*dgcY* | pBBR1MCS-5 contained *dgcY* gene of *H. hydrothermalis* Y2 |
| pBBR1MCS-5-*dgcY_202_****_–_****_406_* | pBBR1MCS-5 contained *dgcY_202_****_–_****_406_* gene of *H. hydrothermalis* Y2 |
| pBBR1MCS-5-*hhmR* | pBBR1MCS-5 contained *hhmR* gene of *H. hydrothermalis* Y2 |

**Table S5** Primers used in this study

| **Name** | **Sequence** | **Application** | |
| --- | --- | --- | --- |
| DgcY-F | CGGGATCCATGTATCGACTTCCCTACTTTC | Constructing protein plasmid | |
| DgcY-R | CCCAAGCTTTAAGGTTGGCTGGGTTCCAATG |  | |
| DgcY*_202_****_–_****_406_*-F | CGGGATCCATGAACACACGCGTAGAG |  | |
| DgcY*_202_****_–_****_406_*-R | CGGGATCCGTGTATCGACTTC |  | |
| DgcY*_222_****_–_****_406_*-F | CGGAATTCATGACCTACCTGGACC |  | |
| DgcY*_222_****_–_****_406_*-R | CCGCTCGAGTAAGGTTGGCTGGG |  | |
| DgcY-mcs5-F | GGAATTCCGTGTATCGACTTCCCTAC |  | |
| DgcY- mcs5-R | CGGGATCCCTATAAGGTTGGCTGG |  |  |
| Δ*dgcY*-1-F | CGGGATCCGGTATTGACGACC | constructing *dgcY* deletion mutant |  |
| Δ*dgcY*-1-R | GTTCTAGACGGGTCCTTGCTCC |  |  |
| Δ*dgcY*-2-F | GGAGCAAGGACCCGTCTAGAAC |  |  |
| Δ*dgcY*-2-R | CCGGAATTCCGCATCGAGGC |  |  |
| GST-DgcY*_202_****_–_****_406_*-1-F | CGCGGATCCCATGTCCCCTATACTAG |  |  |
| GST-DgcY*_202_****_–_****_406_*-1-R | CAATGCGGATTCCATCAGATCCGATTTTGG | Constructing protein plasmid |  |
| GST-DgcY*_202_****_–_****_406_*-2-F | CCAAAATCGGATCTGACACGCGTAGAGCAG |  |  |
| GST-DgcY*_202_****_–_****_406_*-2-R | CCCAAGCTTTAAGGTTGGCTGGGTTC |  | |
| GST-F | GGAATTCATGTCCCCTATACTAGG |  | |
| GST-R | CCCAAGCTTCAGATCCGATTTTG |  | |
| Δ*HhmR* -1-F | CGGAATTCTCATCTCCTGCCCGAACCAG | constructing *hhmR* deletion mutant | |
| Δ*HhmR* -1-R | CAAAGGGAGTGTTGGTTCACGTTCTGCGGG |  | |
| Δ*HhmR* -2-F | CCCGCAGAACGTGAACCAACACTCCCTTTG |  | |
| Δ*HhmR* -2-R | CGGGATCCGCCGGATCACTTGC |  | |
| mcs-5-*HhmR*-F | CGGAATTCGGTGAATCTTGAAACAAAATGG |  | |
| mcs-5-*HhmR*-R | CTGAACCGCCTCCACCTTAGCCCTGCAGAAA |  | |
| *HhmR* -STREP-F1 | CGCGGATCCGTGAATCTTGAAACAAAATGG |  | |
| *HhmR* -STREP-R1 | GTGAGACCATCCACCCGATCCACCGCCCTGCAGAAAAT |  | |
| *HhmR* -STREP-R2 | CCGCTCGAGTTATTTTTCGAACTGAGGGTGAGACCATC |  | |
| *HhmR* -his-F | CGCGGATCCGTGAATCTTGAAAC |  | |
| *HhmR* -his-F | CCGCTCGAGGCCCTGCAGAAAATC |  | |
| 77943-R1 | GGTGAGACCATCCACCCGATCCACCAACGTAATGCGGTGG |  | |
| 77943-R2 | CCGCTCGAGTTATTTTTCGAACTGAGGGTGAGACCATCCAC |  | |
| 77943-F | CGCGGATCCATGAAAAGTACTCTG |  | |
| *HhmR* 1-89-F1 | CGCGGATCCGTGAATCTTGAAAC |  | |
| *HhmR* 1-89-R1 | GTGAGACCATCCACCCGATCCACCAATCGAAAGG |  | |
| *HhmR* 90-305-F1 | CGCGGATCCGCCAACGAGGCGCTGG |  | |
| *HhmR* 90-305-R1 | GTGAGACCATCCACCCGATCCACCGCCCTGCAGAAAATC |  | |
| strep-R | CCGCTCGAGTTATTTTTCGAACTGAGGGTGAGACCATCC |  | |
| P174D-F | GATGTTGGATGTGTCGCTCCCTGATGAGC | Constructing mutated protein plasmid | |
| P174D-R | GCGACACATCCAACATCTTGACCTTGCC |  | |
| D179A-F | GCTCCCTGCAGAGCAGGGCAAGCCGCTG |  | |
| D179A-R | CCTGCTCTGCAGGGAGCGACACGGGCAAC |  | |
| L239R-F | CATGGTCCGCCAAGGGGTCGGCGTGGCCTG |  | |
| L239R-R | CCCCTTGGCGGACCATGCCCTTCAGGCC |  | |
| Q240D-F | GGTCTTGGACGGGGTCGGCGTGGCCTGG |  | |
| Q240D-R | CGACCCCGTCCAAGACCATGCCCTTCAG |  | |
| G259H-F | GAAGAGCCACCGGCTGGTTCGGGCAGGAG |  | |
| G259H-R | CCAGCCGGTGGCTCTTCAACTCTTCACG |  | |
